# Role of Mitochondrial Dynamics in Neuronal Development: Mechanism for Wolfram Syndrome

**DOI:** 10.1371/journal.pbio.1002511

**Published:** 2016-07-19

**Authors:** Michal Cagalinec, Mailis Liiv, Zuzana Hodurova, Miriam Ann Hickey, Annika Vaarmann, Merle Mandel, Akbar Zeb, Vinay Choubey, Malle Kuum, Dzhamilja Safiulina, Eero Vasar, Vladimir Veksler, Allen Kaasik

**Affiliations:** 1 Department of Pharmacology, Institute of Biomedicine and Translational Medicine, University of Tartu, Tartu, Estonia; 2 Department of Muscle Cell Research, Institute of Molecular Physiology and Genetics, Slovak Academy of Sciences, Bratislava, Slovakia; 3 Department of Physiology, Institute of Biomedicine and Translational Medicine, University of Tartu, Tartu, Estonia; 4 INSERM U-1180, Châtenay-Malabry, France; 5 University Paris-Saclay, Châtenay-Malabry, France; National Institutes of Health, UNITED STATES

## Abstract

Deficiency of the protein Wolfram syndrome 1 (WFS1) is associated with multiple neurological and psychiatric abnormalities similar to those observed in pathologies showing alterations in mitochondrial dynamics. The aim of this study was to examine the hypothesis that WFS1 deficiency affects neuronal function via mitochondrial abnormalities. We show that down-regulation of WFS1 in neurons leads to dramatic changes in mitochondrial dynamics (inhibited mitochondrial fusion, altered mitochondrial trafficking, and augmented mitophagy), delaying neuronal development. WFS1 deficiency induces endoplasmic reticulum (ER) stress, leading to inositol 1,4,5-trisphosphate receptor (IP_3_R) dysfunction and disturbed cytosolic Ca^2+^ homeostasis, which, in turn, alters mitochondrial dynamics. Importantly, ER stress, impaired Ca^2+^ homeostasis, altered mitochondrial dynamics, and delayed neuronal development are causatively related events because interventions at all these levels improved the downstream processes. Our data shed light on the mechanisms of neuronal abnormalities in Wolfram syndrome and point out potential therapeutic targets. This work may have broader implications for understanding the role of mitochondrial dynamics in neuropsychiatric diseases.

## Abstract 2

Deficiency of the protein Wolframin in Wolfram syndrome triggers a stress cascade in the endoplasmic reticulum; this leads to altered calcium homeostasis, which in turn impairs mitochondrial dynamics and consequently inhibits neuronal development.

## Author Summary

Wolfram syndrome (WS) is a genetic disorder characterized by diabetes insipidus, diabetes mellitus, optic atrophy, deafness, and brain atrophy. Brain abnormalities occur at the earliest stage of clinical symptoms, suggesting that Wolfram syndrome has a pronounced impact on early brain development. The majority of Wolfram syndrome cases are caused by mutations in the gene Wolfram syndrome 1 (WFS1), which encodes for a protein localized to the endoplasmic reticulum (ER) membrane. However, the clinical symptoms of WS resemble mitochondrial disease symptoms, suggesting strong mitochondrial involvement.

In this manuscript, we demonstrate that deficiency of the gene WFS1 triggers an ER-stress cascade, which impairs the function of the IP_3_-receptor calcium channel, leading to altered calcium homeostasis. The latter leads to dysregulation of mitochondrial dynamics, as characterized by augmented mitophagy—a selective degradation of mitochondria—and inhibited mitochondrial trafficking and fusion, which results in lower levels of ATP and, thus, inhibits neuronal development. These results shed new light onto the mechanisms of neuronal abnormalities in Wolfram syndrome and point out potentially new therapeutic targets. Moreover, our results unravel two rather unexpected links that have an impact beyond the relatively rare Wolfram syndrome. First, relatively mild stress of the ER can seriously disturb mitochondrial dynamics, explaining why alterations at the level of the ER could lead to a mitochondrial phenotype. Second, increased levels of mitophagy, leading to excessive and unwanted mitochondrial clearance, are harmful for neurons. Furthermore, since alterations in the gene WFS1 take place in different neurologic and psychiatric disorders, our work may also have broad implications for understanding the role of mitochondrial dynamics in neuropsychiatric diseases.

## Introduction

Wolfram syndrome (WS) is a genetic disorder characterized by diabetes insipidus, diabetes mellitus, optical atrophy, and deafness (DIDMOAD) and brain atrophy that results in death in middle adulthood, typically due to brainstem atrophy-induced respiratory failure [1]. About 60% of patients with WS develop a neurological or psychiatric disorder, including psychosis, episodes of severe depression, and impulsive and aggressive behaviour. Importantly, brain abnormalities occur at the earliest stage of clinical symptoms, suggesting that WS has a pronounced impact on early brain development [2]. The majority of WS cases are related to mutations in the gene Wolfram syndrome 1 (wolframin, WFS1), which encodes a protein localized in the endoplasmic reticulum (ER) membrane. A number of studies have pointed out the involvement of WFS1 in Ca^2+^ homeostasis and ER stress regulation [3–5]. It has been suggested that the ER stress plays a causative role in WS.

At the same time, the clinical symptoms of WS resemble mitochondrial disease symptoms, such as deafness, optic atrophy, and psychiatric disorders. Moreover, the affected tissues and organs in WS have a high metabolic demand, and most of the clinical manifestations of WS are consistent with an energy metabolism defect. Therefore, another hypothesis has been forwarded that WS is caused by mitochondrial dysfunction [6,7]. This hypothesis is indirectly supported by more recent findings that another causative gene, CISD2, identified in patients with type 2 WS, is associated with mitochondrial abnormalities and activation of mitophagy [8,9].

Importantly, these hypotheses are not mutually exclusive, because ER stress is also capable of impairing mitochondrial function [10]. This rationale further supports the idea that mitochondrial disorders are involved in the pathogenesis of WS.

In the present work, using primary neuronal cultures, we show that WFS1 downregulation leads to marked impairment of mitochondrial dynamics, which, in turn, inhibits neuronal development. We demonstrate that WFS1 deficiency triggers an ER stress associated with inositol 1,4,5-trisphosphate receptor (IP_3_R) dysfunction, leading to altered cell calcium homeostasis. The latter, in turn, is involved in the dysregulation of mitochondrial dynamics (mitophagy and fusion-fission cycle) in neurons. These results shed new light on the mechanisms of neuronal abnormalities in WS and point out potentially new therapeutic targets.

## Results

### WFS1 Deficiency Impairs Mitochondrial Dynamics

**Fig 1:**
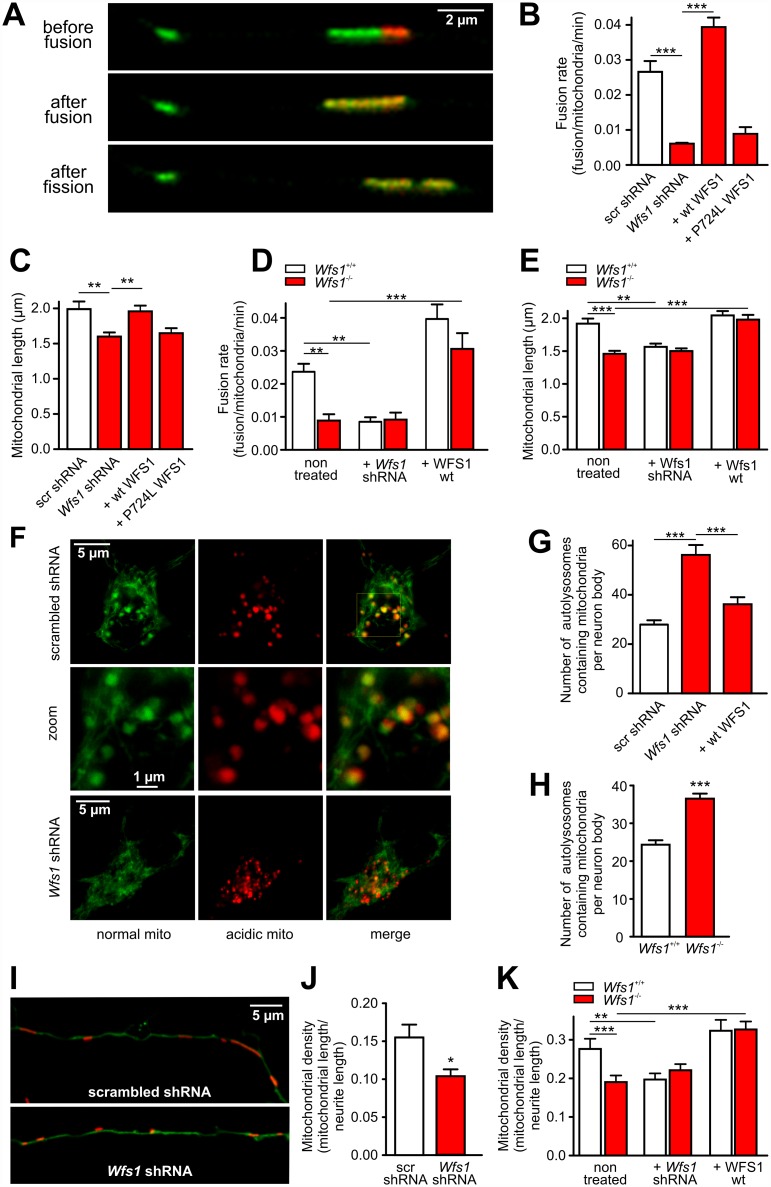
WFS1 deficiency impairs mitochondrial dynamics. (A) Primary cortical neurons were transfected with the photoconvertible mitochondrially targeted construct mito-Kikume-Green and scrambled shRNA or *Wfs1* shRNA. Selected mitochondria were irradiated using a 405-nm laser line, thereby converting mito-Kikume-Green into mito-Kikume-Red. Fusion events between mito-Kikume-Green and photoactivated mito-Kikume-Red mitochondria are visible when mitochondria become yellow after mixing of the contents of the red and green mitochondria. (B–E) In primary cortical neurons, *Wfs1* shRNA significantly decreases fusion rate (B) and mitochondrial length (C). These parameters are restored by overexpression of wild-type (wt) WFS1 but not by P724L WFS1, a mutant found in Wolfram syndrome. Similar changes are observed in cortical neurons isolated from *Wfs1*^-/-^ mice. The lower fusion rate (D) and reduced mitochondrial length (E) in *Wfs*1^-/-^ neurons is restored by wt WFS1 overexpression, but *Wfs1* shRNA has no effect on these parameters. (F–H) Primary cortical neurons were transfected with mitochondrially targeted Keima (which changes its excitation spectrum under acidic conditions) and scrambled shRNA or *Wfs1* shRNA (F). The number of autolysosomes containing mitochondria increases in *Wfs1*-silenced neurons (G) and in neurons isolated from *Wfs1*^-/-^ mice (H). (I–K) Representative images of mitochondrial morphology and density in the axons of scrambled- and *Wfs1*-shRNA transfected neurons (I). The density of axonal mitochondria is reduced in *Wfs1*-silenced neurons (J) and in neurons isolated from *Wfs1*^-/-^ mice (K). This parameter is restored by wt WFS1 overexpression, but *Wfs1* shRNA has no effect. *p < 0.05, **p < 0.01, and ***p < 0.001 compared with respective control groups. Underlying data is shown in S1 Data.

Mitochondrial fusion and fission dynamics were measured using photoconvertible mitochondrially targeted Kikume Green-Red, which enables the quantification of fusion events between green- and red-emitting mitochondria (S1 Video, Fig 1A). There was a significant, 3-fold decrease in the number of fusion events in *Wfs1* shRNA-treated neurons (efficiency of *Wfs1* shRNA is demonstrated in S1 Fig) compared with scrambled shRNA-treated controls (from 0.029 ± 0.001 to 0.010 ± 0.001 fusion/mito/min, respectively, *n* = 80 neurons, *p* < 0.0001; see also Fig 1B, which shows a representative experiment). Also, the mitochondrial fission rate was decreased in *Wfs1* shRNA-treated neurons (from 0.027 ± 0.001 to 0.010 ± 0.001 fission/mito/min, *n* = 80 neurons, *p* < 0.0001), suggesting that WFS1 deficiency greatly prolongs the fusion–fission cycle. These changes in the fusion–fission cycle were associated with a 20% decrease in mitochondrial length (Fig 1C). Overexpression of human shRNA-insensitive wild-type (wt) WFS1 but not P724L mutant (which occurs in WS) restored the mitochondrial fusion dynamics and length in the *Wfs1* shRNA-treated group (Fig 1B and 1C). The inhibition of mitochondrial fusion was also observed in cerebellar granule neurons in which the fusion rate decreased from 0.036 ± 0.006 fusion/mito/min in control to 0.008 ± 0.003 fusion/mito/min in WFS1*-*deficient neurons (*n* = 16 neurons, *p* = 0.001).

We also quantified mitochondrial fusion and fission dynamics in neurons isolated from *Wfs1*^-/-^ and *Wfs1*^+/+^ mice. Of significance, a 2.5-fold decrease in the number of fusion events in neurons from *Wfs1*^-/-^ compared with those from *Wfs1*^+/+^ mice was observed (Fig 1D). Again, this decrease was associated with an approximate 25% decrease in mitochondrial length (Fig 1E). Moreover, *Wfs1* shRNA suppression of fusion rate and induction of mitochondrial shortening in *Wfs1*^+/+^ neurons had no effect in *Wfs1*^-/-^ neurons, thus showing its specificity. In contrast, overexpression of shRNA-insensitive WFS1 restored the mitochondrial fusion dynamics and length in *Wfs1*^-/-^ neurons while having relatively little effect in *Wfs1*^+/+^ neurons (Fig 1D and 1E). No difference, however, was observed between *Wfs1*^-/-^ and *Wfs1*^+/+^ brains in mRNA expression of the main fusion and fission proteins (S2 Fig).

To measure mitophagy, we expressed the mitochondrially targeted pH-dependent protein Keima, the excitation spectrum of which shifts (green to red in Fig 1F) when mitochondria are delivered to acidic lysosomes. The number of autolysosomes containing mitochondria was increased both in wt neurons transfected with *Wfs1* shRNA and in neurons isolated from *Wfs1-*deficient animals (Fig 1G and 1H). Also, the number of mitochondria-containing autophagosomes (LC3-positive dots co-localizing with a mitochondrial marker; S3 Fig) was significantly increased in neuronal bodies of *Wfs1* shRNA-treated neurons (13.8 ± 1.1 versus 8.8 ± 0.8 in scrambled shRNA-transfected neurons, *p* = 0.0004, *n* = 50 neurons). Overexpression of human shRNA-insensitive wt WFS1 in the *Wfs1* shRNA-treated group reduced the mitophagy close to control level (Fig 1G). Note that *Wfs1* silencing enhances LC3-I conversion to LC3-II (S4 Fig), suggesting increased autophagy.

Together, the LC3 and Keima assays demonstrate that WFS1 deficiency increases mitochondrial removal (mitophagic flux) rather than inhibiting the formation of autolysosomes containing mitochondria. Importantly, WFS1-deficient neurons showed fewer mitochondria in axons. Analysis of mitochondrial density in neurites revealed an approximate 30% decrease in mitochondrial mass both for *Wfs1* shRNA-treated neurons and for neurons isolated from *Wfs1*^-/-^ mice (Fig 1I–1K). This effect was phenocopied by *Wfs1* shRNA in *Wfs1*^+/+^ neurons and rescued by wt WFS1 overexpression in *Wfs1*^-/-^ neurons.

Mitochondrial trafficking was also disturbed in WFS1-deficient neurons. Mitochondria in these neurons showed a decrease in velocity in both antero- and retrograde motion (Table 1, S5 Fig). Mitochondria from WFS1-deficient neurons also changed their direction during motion more often and made more individual runs, whereas the length of these runs was shorter. These data explain the oscillatory-like movement we frequently observed in WFS1-deficient neurons (S2 and S3 Videos). We also measured the contact rate of mitochondria as an indirect parameter of mitochondrial movement. This parameter was also significantly reduced in neurons isolated from *Wfs1*-deficient mice and restored by WFS1 overexpression (S6 Fig).

**Table 1:** Parameters of mitochondrial trafficking in neurons transfected with scrambled shRNA or *Wfs1* shRNA.

Motility parameter	Scrambled shRNA	*Wfs1* shRNA	*P*-value
Fraction of time in motion	0.39 ± 0.01 (0.31)	0.40 ± 0.01 (0.33)	0.3932
Velocity when in motion (μm/s)	1.41 ± 0.09 (0.65)	1.14 ± 0.12 (0.59)	0.0047
Velocity when in anterograde motion (μm/s)	1.18 ± 0.08 (0.64)	0.95 ± 0.10 (0.60)	0.0107
Velocity when in retrograde motion (μm/s)	1.11 ± 0.08 (0.60)	0.95 ± 0.09 (0.56)	0.0358
Change of direction during motion (turn/min)	0.37 ± 0.02 (0.20)	0.62 ± 0.04 (0.42)	0.0001
Average length of anterograde run (μm)	4.10 ± 0.66 (0.71)	2.64 ± 0.75 (0.71)	0.0422
Average length of retrograde run (μm)	3.22 ± 0.55 (0.71)	2.68 ± 0.62 (0.71)	0.1439
Number of anterograde runs (run/min)	0.71 ± 0.02 (0.67)	0.83 ± 0.03 (0.80)	0.0001
Number of retrograde runs (run/min)	0.70 ± 0.03 (0.62)	0.81 ± 0.03 (0.81)	0.0015

Data are presented as the mean ± SEM (median is shown in brackets, *n* = 383 for scrambled shRNA and *n* = 327 for *Wfs1* shRNA, Mann-Whitney test).

### WFS1 Deficiency Is Associated with Decreased Mitochondrial Membrane Potential and Cellular ATP

**Fig 2:**
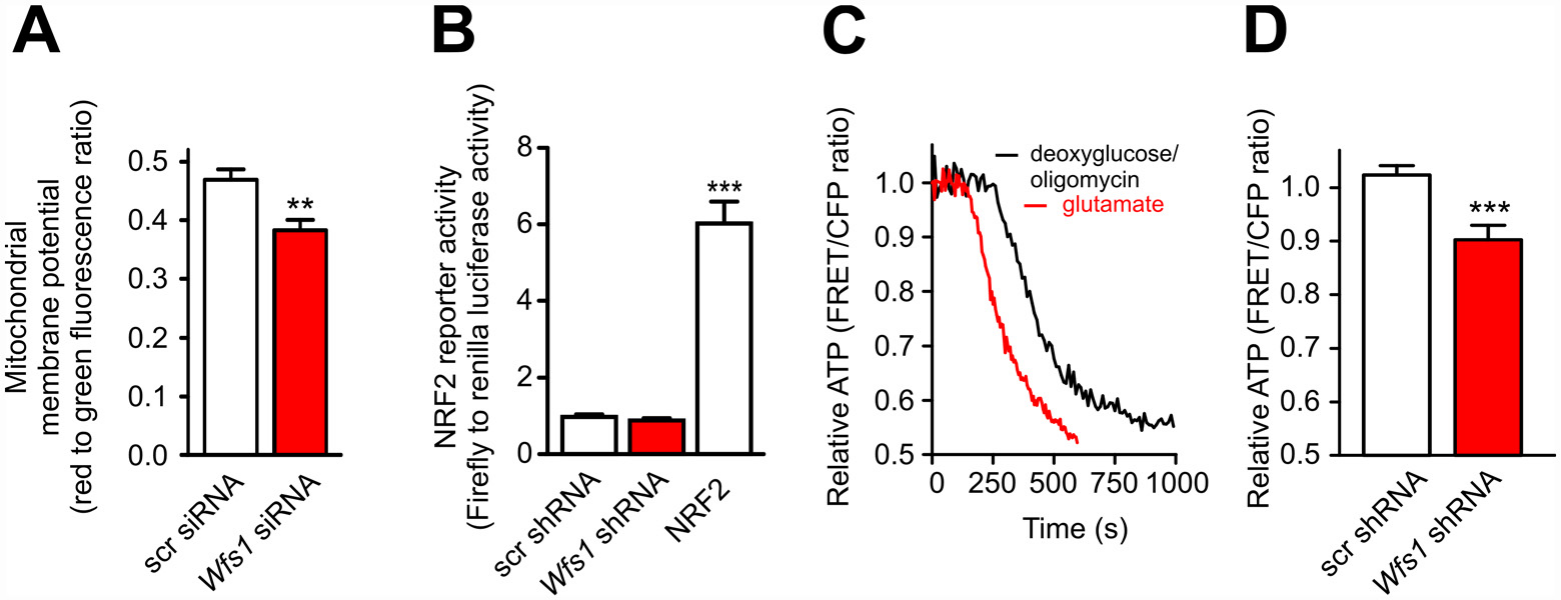
WFS1 deficiency decreases mitochondrial membrane potential and cytosolic ATP level. (A) Primary cortical neurons were transfected with control or *Wfs1* siRNA using the N-TER nanoparticle siRNA transfection system and stained with JC-10, which emits light from 525 nm to 590 nm, depending on mitochondrial membrane potential. Values shown are corrected by subtracting the values obtained in the presence of FCCP incubation (5 μM). The red to green fluorescence ratio demonstrated a slight but significant decrease in the *Wfs1* siRNA group. (B) Neurons were transfected with plasmids expressing scrambled shRNA or *Wfs1* shRNA, firefly luciferase construct containing NRF2 binding site, and *Renilla* luciferase. Firefly luciferase signal normalized to *Renilla* signal demonstrates no change in NRF2 activity. NRF2 overexpression-induced reporter activity was used as a positive control. (C) Neurons transfected with the ATP sensor ATeam and treated with 2-deoxyglucose (12 mM)/oligomycin (2.5 μM) or glutamate (2 mM) (both used as positive controls) show a decrease in relative cytosolic ATP levels. (D) Neurons were transfected with the ATP sensor ATeam and scrambled or *Wfs1* shRNAs. WFS1-deficient neurons show a lower cytosolic ATP level as compared to control. **p < 0.01 and ***p < 0.001 compared with respective control group. Underlying data is shown in S1 Data.

We performed a quantitative analysis of mitochondrial membrane potential using the ratiometric mitochondrial membrane potential-sensitive fluorescent probe JC-10 (emitting light from 525 nm to 590 nm depending on mitochondrial membrane potential). Neurons were first transfected with *Wfs1* siRNA using the N-TER nanoparticle siRNA transfection system to ensure >70% transfection efficiency. The results obtained demonstrated a 10% decrease in red to green fluorescence ratio, suggesting a slight depolarisation in the *Wfs1* siRNA group (Figs 2A and S7). Interestingly, an increased number of polarised, tetramethylrhodamine ethyl ester (TMRE)-positive mitochondria were observed inside autophagosomes in WFS1-deficient neurons (S8 Fig); this suggests that WFS1 deficiency may induce mitophagy of active, polarised mitochondria. We also performed indirect reactive oxygen species (ROS) measurements using an NRF2 reporter gene assay, which showed no difference between the scrambled and *Wfs1* shRNA-transfected neurons (Fig 2B).

Next, we estimated cell ATP levels using the fluorescence resonance energy transfer (FRET)-based ATP sensor ATeam, which employs the epsilon subunit of a bacterial F_0_F_1_-ATPase. Control experiments with deoxyglucose/oligomycin or glutamate reduced neuronal ATP levels and demonstrated the expected decline in FRET signal, showing the validity of our approach (Fig 2C). The results obtained (Fig 2D) show that the cellular ATP level is reduced in WFS1-deficient neurons.

### Impairment of Mitochondrial Dynamics Is Linked to WFS1 Deficiency-Induced Mild ER Stress

**Fig 3:**
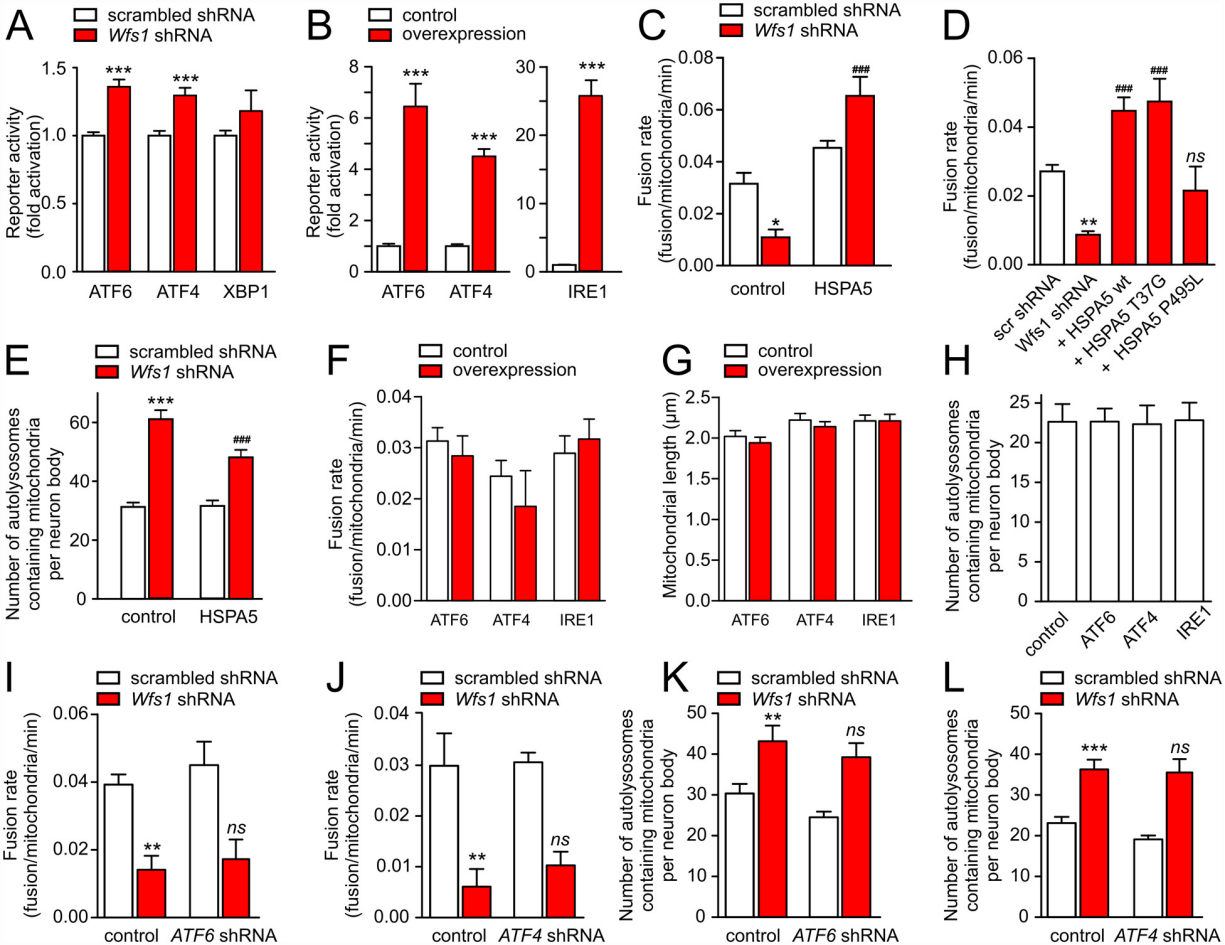
WFS1 deficiency induces mild ER stress in primary cortical neurons. (A) Neurons were transfected with plasmids expressing scrambled shRNA or *Wfs1* shRNA, firefly luciferase constructs containing ATF6 or ATF4 binding sites or a XBP-1 splicing reporter, and *Renilla* luciferase. Firefly luciferase signal normalized to *Renilla* signal demonstrates a moderate increase in ATF6 and ATF4 reporter activity. (B) Positive control experiments in which the above-mentioned reporter systems and *Renilla* luciferase were co-transfected with ATF4, ATF6, or IRE1. (C) The mitochondrial fusion rate is reduced by *Wfs1* shRNA and is restored by co-expressing wt HSPA5 (*p* = 0.001 for interaction, two-way ANOVA). (D) ATPase-deficient HSPA5 mutant (T37G) but not peptide binding-deficient mutant (P495L) restores the fusion rate reduced by *Wfs1* shRNA. (E) HSPA5 overexpression attenuates mitophagy activated by *Wfs1* silencing (*p* = 0.012 for interaction). (F–H) Activation of the primary ER stress pathways by overexpression of ATF6, ATF4, or IRE1 modulates neither fusion rate (F), mitochondrial length (G), nor mitophagy (H). (I–L) Silencing of *ATF6* or *ATF4* modulates neither fusion rate (I, J) nor mitophagy (K, L). *p < 0.05, **p < 0.01, and ***p < 0.001 compared with respective control groups, or ^###^*p* < 0.001 compared with the *Wfs1* shRNA-transfected control group and ^ns^ non-significant compared with the *Wfs1* shRNA-transfected control group. Underlying data is shown in S1 Data.

It has been previously demonstrated that WFS1 deficiency leads to ER stress in different rodent and human cell lines [5], WFS1-deficient β-cells [11], and WFS1-deficient mouse retinas [12]. We checked whether the suppression of WFS1 in neurons also induced ER stress. Using reporter constructs for ATF6, IRE1-XBP1, and PERK-ATF4 pathways, we demonstrate (Fig 3A) that WFS1 suppression activates luciferase reporter constructs with a promoter containing ATF6 and ATF4 binding sites, but not the XBP1 splicing reporter. This WFS1 deficiency-induced activation, however, was weak when compared with that induced by overexpression of ATF6, ATF4, and IRE1 (Fig 3B).

To test whether the effect of WFS1 deficiency on mitochondrial dynamics is associated with ER stress, we mitigated ER stress by overexpressing the ER chaperon HSPA5. Fig 3C and 3D demonstrate that wt HSPA5 and its ATPase-deficient mutant (T37G), but not its protein binding-defective mutant (P495L), restore the *Wfs1* shRNA-suppressed fusion rate. Also, wt HSPA5 partially suppressed WFS1 deficiency-induced mitophagy (Fig 3E).

Importantly, activation of major ER stress-response pathways by overexpressing ATF6, ATF4, or IRE1 neither inhibited mitochondrial fusion nor induced mitochondrial shortening or mitophagy (Fig 3F–3H). Furthermore, *ATF4* and *ATF6* shRNAs were not able to restore mitochondrial fusion rate or decrease mitophagy in WFS1-deficient neurons (Fig 3I–3L). These data suggest that although WFS1 deficiency-induced mild ER stress is followed by disturbed mitochondrial dynamics, the latter is not mediated by these major ER stress-response pathways.

### IP_3_R-Controlled Calcium Homeostasis Is Impaired in WFS1-Deficient Neurons

**Fig 4:**
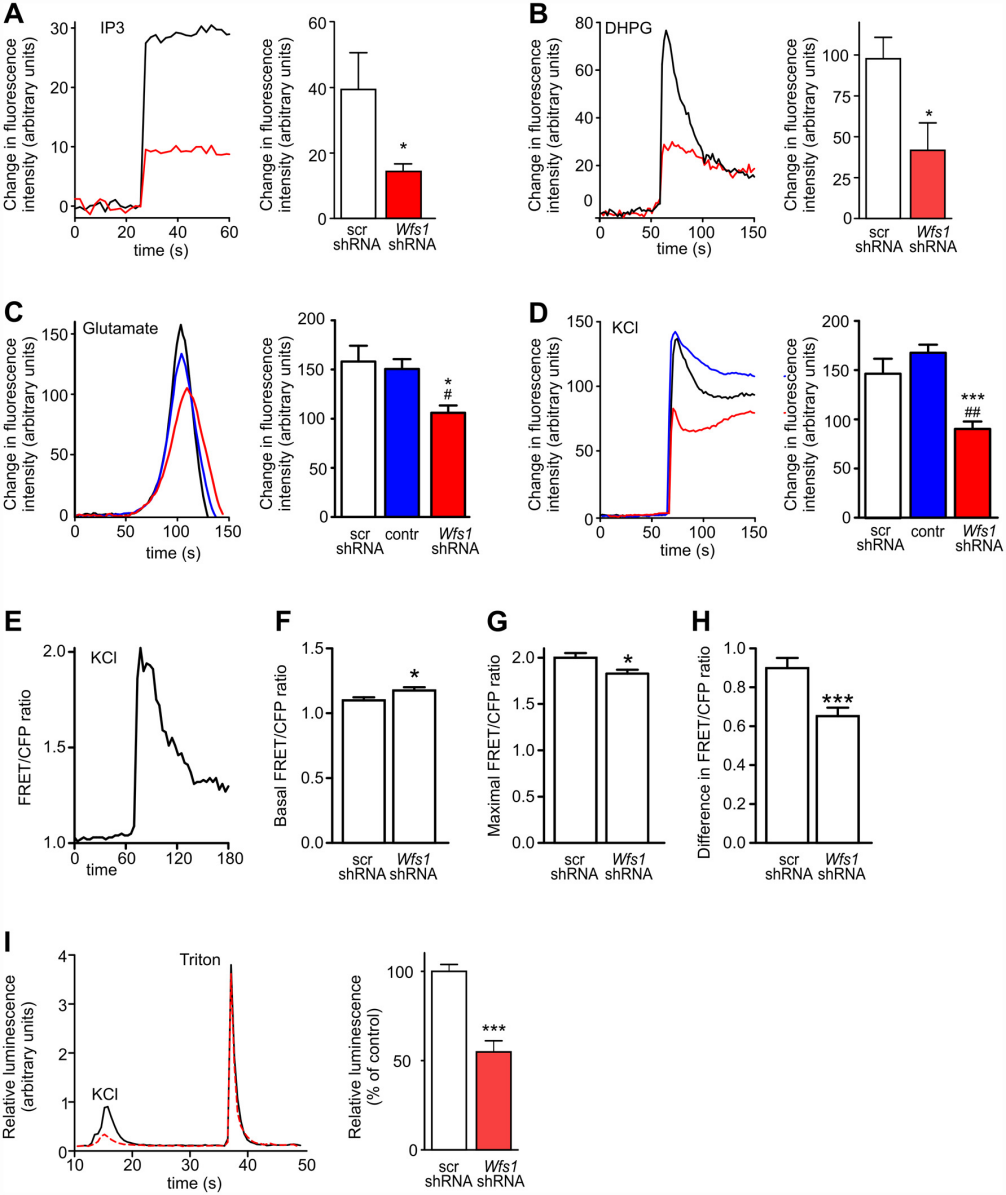
WFS1 deficiency leads to impaired IP_3_R-mediated ER calcium release and disturbed Ca^2+^ homeostasis in primary cortical neurons. (A–D) Neurons transfected with the mitochondrial marker mKate2-mito, scrambled shRNA (black line), or *Wfs1* shRNA (red line) were loaded with the Ca^2+^ sensor Fluo-4. The left panels show time-dependent responses of cytosolic Ca^2+^ to various challenges, and the right panels show the mean changes in fluorescence intensity. Cells were co-loaded with the membrane-permeant caged derivative of IP_3_, and the latter was uncaged by irradiating individual cells with a 405-nm laser (A). Cytosolic Ca^2+^ transients were induced by 200 μM DHPG (B), 2 mM glutamate (C), or 25 mM KCl (D). Graphs depicting glutamate- and KCl-induced Ca^2+^ transients also include an additional control group (blue line) when non-transfected neurons in *Wfs1* shRNA experiments were analysed. (E–H) Cytosolic Ca^2+^ transients (E) were elicited by 25 mM KCl in neurons transfected with the FRET-based cytosolic Ca^2+^ sensor cytoD3cpv and scrambled shRNA or *Wfs1* shRNA. In WFS1-deficient neurons, basal cytosolic Ca^2+^ is higher (F) and stimulated maximal cytosolic Ca^2+^ is lower (G) so that the amplitude of Ca^2+^ transient decreases (H). (I) Neurons were transfected with aequorin and with scrambled shRNA (black line) or *Wfs1* shRNA (red line). Cytosolic Ca^2+^ transients were triggered by 100 mM KCl, after which the cells were lysed with Triton X-100 to measure maximal activity of aequorin. The left panel demonstrates the time course of luminescence change; the right panel shows relative luminescence values (KCl to Triton X-100 ratio) normalised to control conditions. *p < 0.05 and ***p < 0.001 compared with scrambled shRNA, or ^#^*p* < 0.05 and ^##^*p* < 0.01 compared with the non-transfected control group. Underlying data is shown in S1 Data.

An earlier report demonstrated that ER stress-impaired IP_3_R-mediated Ca^2+^ release from the ER [13]. It is noteworthy to mention that IP_3_R rather than ryanodine receptors are primarily responsible for Ca^2+^ release in cortical neurons (S9A Fig). To test whether IP_3_R-mediated Ca^2+^ homeostasis is impaired in WFS1-deficient neurons, we loaded neurons with both the Ca^2+^ sensor Fluo-4 and a membrane-permeant caged derivative of IP_3_. IP_3_ uncaging-induced Ca^2+^ release from the ER to cytosol was notably decreased in WFS1-deficient neurons (Fig 4A). Similarly, the selective group I metabotropic glutamate receptor agonist dihydroxyphenylglycine (DHPG), which stimulates phospholipase C and promotes endogenous IP_3_ formation, induced diminished cytosolic Ca^2+^ transients in WFS1-deficient neurons (Fig 4B). No difference was observed in basal ER [Ca^2+^] (S10 Fig) or in maximal ER Ca^2+^ uptake capacity (S9B Fig), suggesting that the decreased IP_3_-dependent Ca^2+^ release was not due to reduced ER Ca^2+^ levels. Also, no difference was observed in the cytosolic Ca^2+^ transients elicited by an inhibitor of endoplasmic reticulum Ca^2+^ ATPase, 30 μM cyclopiazonic acid (CPA), suggesting that there is no difference in releasable ER Ca^2+^ between control and *Wfs1* silenced neurons (S9C and S10D Figs).

It has been proposed that the IP_3_ receptor is involved in Ca^2+^-induced Ca^2+^ release from the ER (for review, see [14]). Thus, it is reasonable to suggest that impaired IP_3_R function would affect Ca^2+^ release from the ER during neuronal depolarisation. Indeed, by following changes in cytosolic Fluo-4 fluorescence, we observed that cytosolic Ca^2+^ transients in neurons in response to glutamate or KCl were up to 2-fold lower in WFS1-deficient neurons when compared with control groups (Fig 4C and 4D).

**Fig 5:**
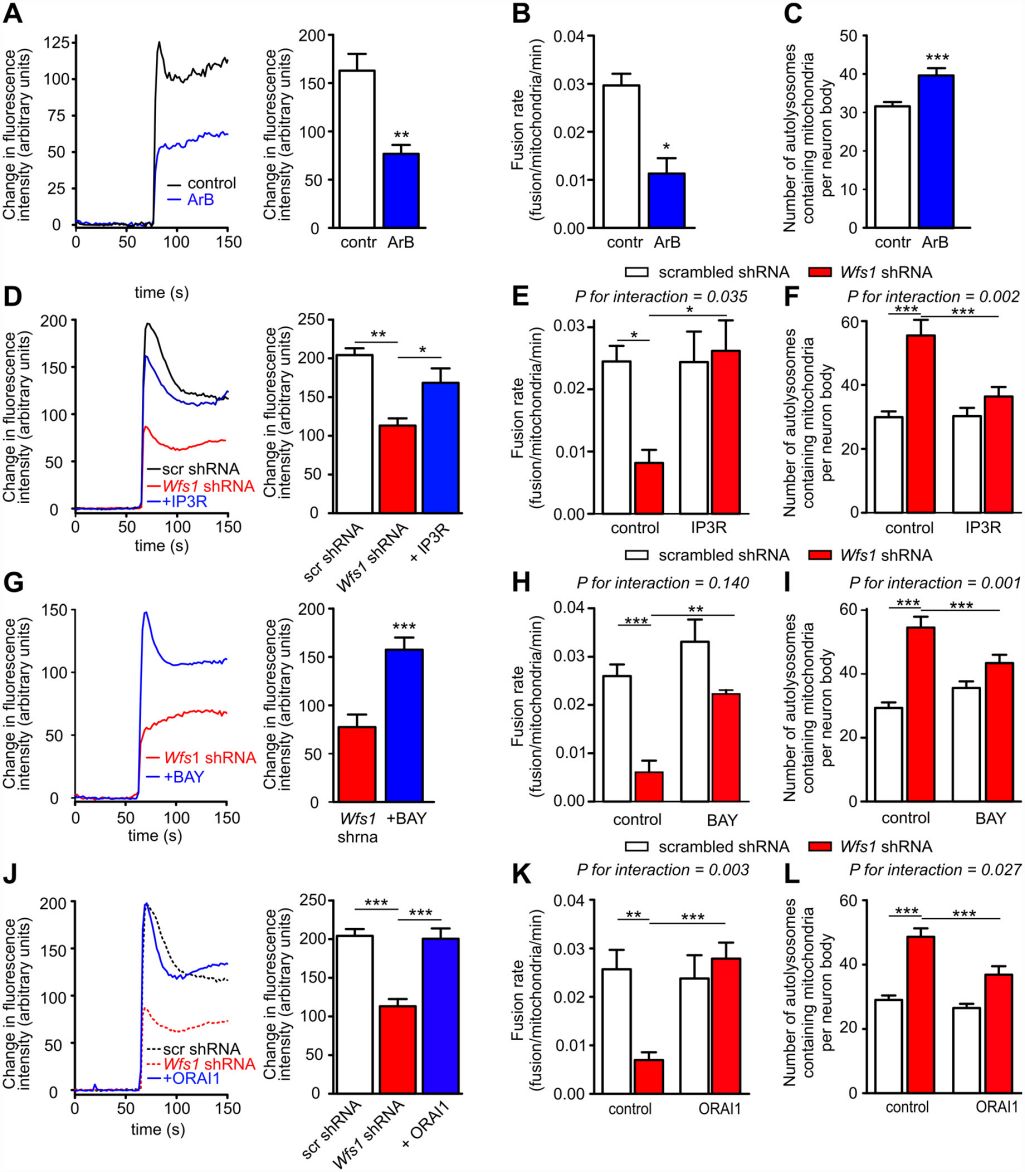
Cytosolic Ca^2+^ homeostasis regulates the mitochondrial fusion rate and mitophagy in primary cortical neurons. (A–C) The IP_3_R inhibitor Araguspongin B (5 μM) decreases KCl-induced Ca^2+^ transients (A), reduces the mitochondrial fusion rate (B), and increases mitophagy in control neurons (C). For D–L, neurons were transfected with the mitochondrial marker mKate2-mito and scrambled shRNA (white bars) or *Wfs1* shRNA (red bars). (D–F) Under conditions of WFS1 deficiency, IP_3_R overexpression restores the depressed cytosolic Ca^2+^ response to 25 mM KCl (D) and normalises the mitochondrial fusion rate (E) and mitophagy (F). (G–I) Similarly, under conditions of WFS1 deficiency, the L-type Ca^2+^ channel agonist Bay K 8644 (5 μM) increases the cytosolic Ca^2+^ response to KCl (G) and normalises the mitochondrial fusion rate (H) and mitophagy (I). (J–L) Overexpression of ORAI1 has similar effects (dotted lines in J represent responses of cells treated with scrambled or *Wfs1* shRNAs already shown in D). *p < 0.05, ** *p* < 0.01, and *** *p* < 0.001 versus indicated groups. *P*-values for interactions are given in the figures. Underlying data is shown in S1 Data.

We next tested whether decreased IP_3_-dependent Ca^2+^ release is associated with altered basal cytosolic [Ca^2+^] levels. We used a FRET-based Ca^2+^ sensor to enable measurement of cytosolic [Ca^2+^] before and after KCl treatment. The data obtained demonstrated increased basal [Ca^2+^] in WFS1-deficient neurons, whereas stimulation led to lower maximal [Ca^2+^] (Fig 4E–4G). Accordingly, the amplitude of Ca^2+^ transient was significantly decreased (Fig 4H). Similarly, a decrease was observed when aequorin-emitted bioluminescence was used to quantify the maximal [Ca^2+^] after stimulation (Fig 4I). Furthermore, pre-treatment with Araguspongin B, an inhibitor of IP_3_-dependent Ca^2+^ release, suppressed KCl-induced cytosolic Ca^2+^ transients in wt neurons (Fig 5A), whereas overexpression of the active fragment of IP_3_R restored the cytosolic Ca^2+^ transients in WFS1-deficient neurons (Fig 5D).

These findings suggest that WFS1 deficiency induces lower IP_3_R-mediated Ca^2+^ release.

### Disturbed Calcium Homeostasis Is Responsible for Impaired Mitochondrial Dynamics in WFS1-Deficient Neurons

Next, we checked whether the disturbed cytosolic Ca^2+^ homeostasis could be responsible for the impaired mitochondrial dynamics observed in WFS1-deficient neurons. Indeed, IP_3_R inhibition by Araguspongin B suppressed the mitochondrial fusion rate and initiated mitophagy in wt neurons (Fig 5B and 5C). In contrast, overexpression of the active fragment of IP_3_R corrected the WFS1 deficiency-induced changes in mitochondrial dynamics (Fig 5E and 5F). The latter suggests a direct link between IP_3_R-mediated ER calcium release and mitochondrial dynamics.

Furthermore, treatment of WFS1-deficient neurons with the L-type Ca^2+^ channel activator, Bay K 8644, restored cytosolic Ca^2+^ transients (Fig 5G), restored the fusion rate, and inhibited mitophagy (Fig 5H and 5I). A similar rescue was obtained by overexpressing plasma membrane ORAI calcium release-activated calcium modulator 1 (ORAI1), which also corrected cytosolic Ca^2+^ transients, restored the fusion rate, and suppressed mitophagy in WFS1-deficient neurons (Fig 5J–5L). These experiments suggest that disturbed cytosolic Ca^2+^ homeostasis, rather than ER Ca^2+^ specifically, or direct ER-mitochondria Ca^2+^ channelling, is responsible for impaired mitochondrial dynamics in WFS1-deficient neurons.

### WFS1 Deficiency Delays Neuronal Development and Impairs Neuronal Survival

**Fig 6:**
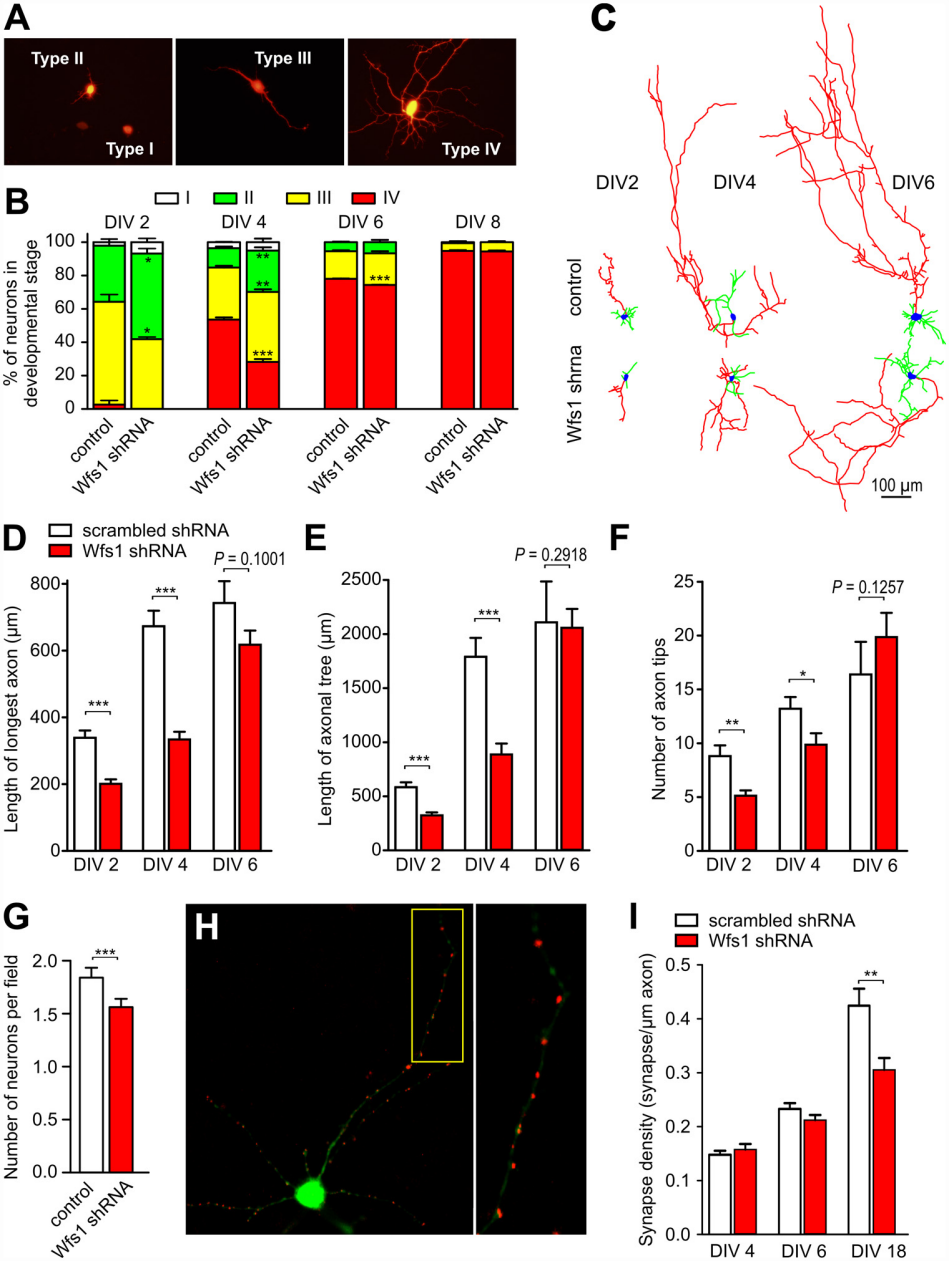
WFS1 deficiency leads to impaired neuronal development. Primary cortical neurons were transfected with the neuronal marker pAAV-hSyn-DsRed1 and scrambled shRNA or *Wfs1* shRNA at DIV (day in vitro) 1, and neuronal morphology was assessed at different time points. (A) Cell morphology at different stages of development. (B) Morphological analysis demonstrating the retarded development of WFS1-deficient neurons. (C) Examples of reconstructed control and WFS1-deficient neurons at different DIV. (D–F) WFS1 deficiency retards growth of the longest axon (D) and growth of the axonal tree (E) and decreases the number of axon tips (F). (G) Survival of WFS1-deficient neurons is decreased when compared with control neurons (*n* = 61–62 individual dishes from 17 independent sister cultures at DIV 6–11). (H) Visualisation of synapses (red) using an antibody targeted against the post-synaptic marker PSD-95 in neurons transfected with GFP (green). The right panel shows a zoomed image. (I) WFS1-deficiency decreases synaptic density at late stages. *p < 0.05, **p < 0.01, and ***p < 0.001 compared with respective scrambled shRNA-treated groups. Underlying data is shown in S1 Data.

**Fig 7:**
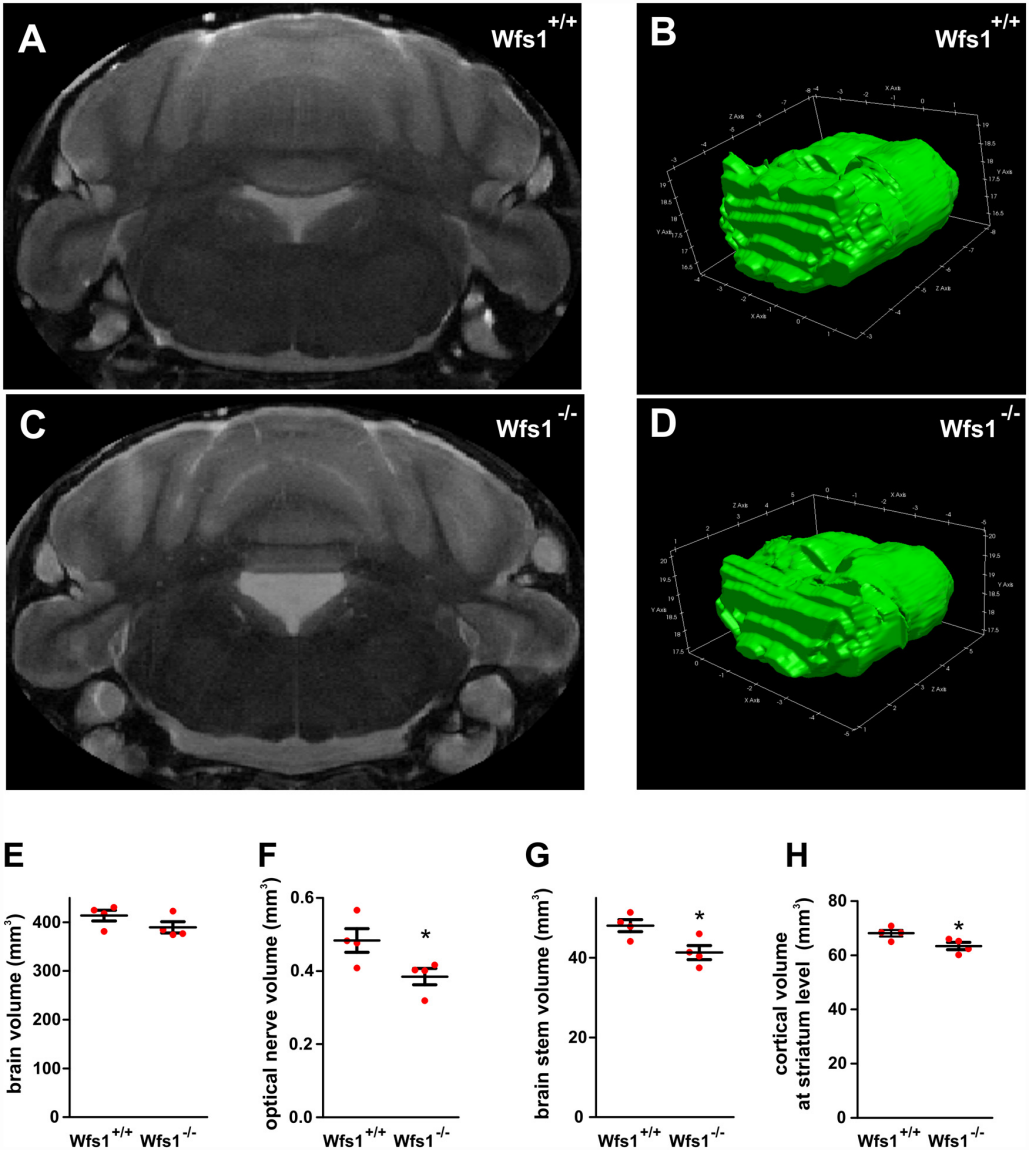
WFS1 deficiency is associated with reduced volume of the optic nerve, brain stem, and cortex at the level of the striatum. (A–D) Brains, within skulls, were scanned *ex vivo* using a 94/20 Bruker BioSpec MRI. Representative single coronal slice from the imaging sequence (A, C) and a 3D reconstruction of the brain stem (B, D). The outer skull, tissue, and surrounding medium have been removed for clarity. (E–H) Volumetric analysis of brains from *Wfs* ^+/+^ and ^-/-^ male mice: whole brain (E), optic nerve (F), brain stem (G), and cortical volumes at the level of striatum (H). *p < 0.05 and **p < 0.01 compared with *Wfs* ^+/+^, groups (*n* = 4 animals in each group).

Because WS has been shown to be associated with both neurodegeneration and impaired early brain development [2], we further aimed to test whether the alterations in Ca^2+^ homeostasis and mitochondrial dynamics affect neuronal development or/and survival. WFS1 deficiency delayed the development of cortical neurons markedly (Fig 6A and 6B; see S1 Table for original data). The longest axon and the axonal tree were significantly shorter, and the number of axonal tips was lower in developing DIV2-DIV4 *Wfs1* shRNA-transfected neurons (Fig 6C–6F). However, in relatively mature neurons, at DIV6, the axonal length and branching was similar to control. Fig 6G demonstrates that the survival of WFS1-deficient neurons is also impaired; transfection of *Wfs1* shRNA led to relatively slight but significant loss of neurons. Interestingly, WFS1 suppression decreased significantly the density of synapses when measured at DIV19 but not at earlier stages (Fig 6H and 6I). To examine whether the compromised development and survival we observed in vitro have relevance in vivo, we further conducted ex vivo magnetic resonance imaging of the brains of 1-y-old *Wfs1*-deficient male mice. Volumetric analysis did not demonstrate a significant change in total cerebral volume, but there was a marked reduction of the optic nerve and brain stem volumes in *Wfs1* deficient mice (Fig 7A–7G). A slight decrease was also observed in cortical area at the level of striatum (Fig 7H). Thus, these results suggest that WFS1 is indispensable for appropriate neuronal development, morphology, and survival both in vitro and in vivo.

### Correction of IP3R Function and Mitophagy Rescues Developmental Delay in WFS1 Deficiency

**Fig 8:**
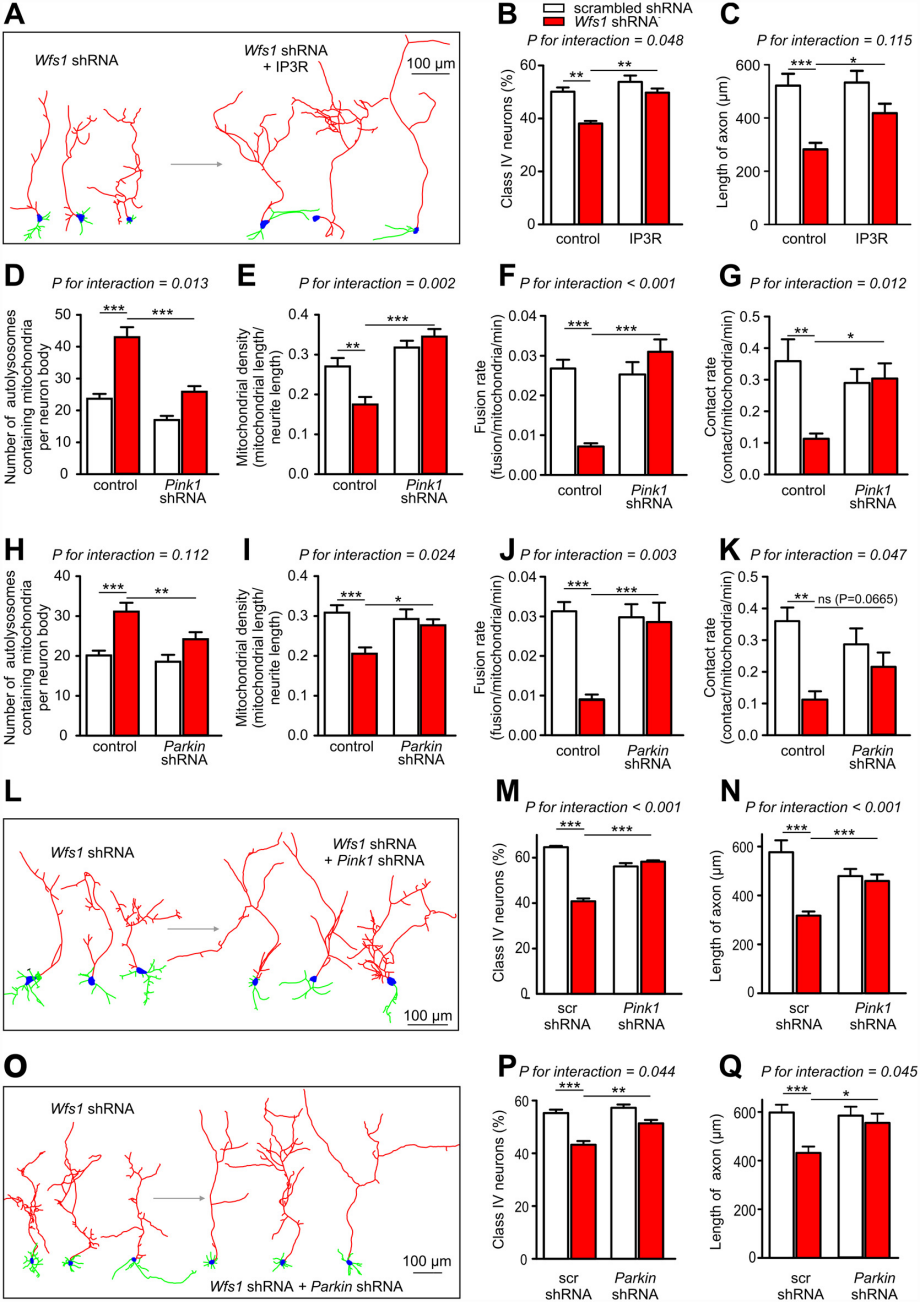
Overexpression of IP_3_R or blocking mitophagy rescues impaired neuronal development in Wfs1 deficiency. (A–C) Examples of reconstructed WFS1-deficient neurons co-transfected with IP_3_R (A). Under conditions of WFS1 deficiency, overexpression of IP_3_R normalises the percentage of mature neurons (stage IV) at DIV4 (B) and axon lengths (C). (D–G) Pink1 silencing normalises mitophagy (D), mitochondrial density (E), fusion (F), and contact rate (G). (H–K) Parkin silencing shows similar effects on all these parameters. (L–N) Examples of reconstructed WFS1-deficient neurons co-transfected with *PINK1* shRNA (L). *PINK1* silencing normalises the percentage of mature neurons (stage IV) at DIV4 (M) and axon lengths (N). Similarly, *Parkin* silencing corrects neuronal maturation and axonal growth (O–Q). White bars = scrambled shRNA, red bars = *Wfs1* shRNA. * *p* < 0.05, ** *p* < 0.01, and *** *p* < 0.001 versus indicated group. *P-*values for interactions are given in the figure. Underlying data is shown in S1 Data.

We next tested the possibility that delayed neuronal development in WFS1 deficiency is a consequence of IP_3_R dysfunction and/or impaired mitochondrial dynamics. If this hypothesis is correct, it could open the possibility of improving neuronal development by restoring cytosolic Ca^2+^ homeostasis or mitochondrial dynamics. Indeed, overexpression of IP_3_R improved Ca^2+^ homeostasis, protected against WFS1 deficiency-induced developmental delay and also partially restored axonal growth (Fig 8A–8C). Importantly, this overexpression did not suppress ER stress (S11 Fig), thus suggesting that IP_3_R-mediated Ca^2+^ disturbances rather than ER stress per se are responsible for the neuronal development delay. Furthermore, overexpression of ORAI1 normalized cytosolic Ca^2+^ homeostasis and also protected neurons against the development delay (S12 Fig).

Finally, we tested whether specific suppression of mitophagy in WFS1 deficiency by co-expression of *Wfs1* shRNA with *Pink1* or *Parkin* shRNAs could rescue the neuronal development. Both shRNAs suppressed effectively the WFS1 deficiency-induced mitophagy and restored mitochondrial density (Fig 8D, 8H, 8E and 8I), demonstrating that WFS1 deficiency activates selective Pink1-Parkin-dependent mitophagy. The latter suggestion is supported by the finding that Parkin translocation to mitochondria was higher in *Wfs1* shRNA-expressing PC6 cells, PC12 cells (S13 Fig), and in neurons (S14 Fig). Moreover, *Pink1* and *Parkin* shRNAs restored the fusion rate and contact rate (an indirect parameter for mitochondrial movement; Fig 8F, 8J, 8G and 8K), suggesting the relevance of mitophagy in these processes. Most importantly, suppression of mitophagy by expressing *Pink1* or *Parkin* shRNAs accelerated development and restored the axonal growth in WFS1-deficient neurons (Fig 8L–8Q). At the same time, *Parkin* and *Pink1* shRNAs neither suppressed WFS1 deficiency-induced ER stress nor restored Ca^2+^ transients (S15 Fig), suggesting that the impairment of mitochondrial dynamics is a downstream event relative to the Ca^2+^ homeostasis disturbances and that overactivated mitophagy is the primary reason for delayed neuronal development in WFS1-deficient neurons.

We also tested whether the inhibition of mitochondrial fission proteins could protect mitochondria in WFS1-deficient neurons and rescue the neurons from the developmental delay. Treatment with negative dominant DRP1 (nd DRP1) reversed the negative effects of WFS1 deficiency on fusion and density loss and restored normal development (S16A–S16C Fig). nd DRP1 also protected against the inhibition of mitochondrial fusion induced by the ER stressor Brefeldin A (S16D Fig). Brefeldin A itself showed too strong a negative effect on neuronal survival, making it impossible to estimate neuronal development.

## Discussion

Our results uncover a chain of causal links relating ER stress, cytosolic Ca^2+^ disturbances, impaired mitochondrial dynamics, and delayed neuronal development in WFS1-deficient neurons. We demonstrate that WFS1 deficiency induces ER stress in neurons (as has already been shown in other cell types [5,15]), affecting the IP_3_R receptor. This was associated with higher cytosolic Ca^2+^ at resting conditions (which is consistent with previously reported elevated basal cytosolic [Ca^2+^] in Wfs1 deficient iPS cells [16]) but lower maximal [Ca^2+^] under stimulated conditions, suggesting reduced amplitude of IP_3_R-mediated Ca^2+^ release in WFS1-deficient neurons. We show that the amplitude of IP_3_R-mediated Ca^2+^ release induced by photolysis of caged IP_3_ or by activating endogenous IP_3_ production by the metabotropic glutamate receptor agonist DHPG was significantly lower in WFS1-deficient neurons. The exact mechanism of this WFS1-dependent IP_3_R dysfunction is not clear; however, it has been previously shown that ER stress induced IP_3_R inhibition by impairing the IP_3_R-HSPA5 interaction [13].

We further demonstrate that altered Ca^2+^ homeostasis disturbs mitochondrial dynamics. Mitochondria in WFS1-deficient neurons do not move properly, they do not fuse and split apart as frequently as their wt counterparts, and they undergo mitophagy more frequently. Overexpression of the active IP_3_R fragment restores IP_3_R-mediated Ca^2+^ release and corrects all perturbations in mitochondrial dynamics, suggesting that these events are causally linked. Pharmacological inhibition of IP_3_R by Araguspongin B phenocopied the effects of WFS1 deficiency, confirming the connection between reduced ER Ca^2+^ release and impaired mitochondrial dynamics. Importantly, we were able to correct mitochondrial dynamics by activating store*-*operated calcium entry or by activating L-type Ca^2+^ channels pharmacologically, linking the lower ER Ca^2+^ release with impaired mitochondrial dynamics. Potentially, there are several ways how ER Ca^2+^ release could influence mitochondrial dynamics. Lowered levels of ER Ca^2+^ release could directly activate/deactivate mitochondrial and/or cytoskeleton proteins involved in mitochondrial dynamics. One may suggest that Ca^2+^ affects activity or expression of these proteins through the calcium/calmodulin (CaM) kinase signalling cascade, which may not be sufficiently activated in WFS1 deficiency. This question deserves specific further study.

We also demonstrate that the mechanism linking WFS1 deficiency-related ER stress with impaired mitochondrial dynamics involves two Parkinson’s disease-related proteins, PINK1 and Parkin. Both *Pink1* and *Parkin* shRNAs supressed WFS1 deficiency-induced mitophagy back to control levels. These data suggest that WFS1 deficiency may activate the PINK1 and Parkin pathway (supported by our finding demonstrating increased Parkin translocation to mitochondria under basal conditions), which has been shown to inhibit mitochondrial movement [17] and fusion-fission dynamics [18–20] and induce mitophagy [21–24]. Importantly, *Pink1* and *Parkin* shRNAs also restored mitochondrial fusion–fission dynamics and trafficking, suggesting activation of PINK1-Parkin pathway to be the primary event leading to impaired trafficking and fusion rate as well as to mitophagy. The result that PINK1 or Parkin silencing (which improves mitochondrial dynamics in WFS1 deficient neurons) does not correct ER stress Ca^2+^ responses in WFS1 deficiency suggests that regulation of mitochondrial dynamics by the PINK1-Parkin pathway is downstream to cytosolic Ca^2+^ homeostasis.

In principle, there are two potential explanations for how PINK1-Parkin pathway could be involved. First, mitochondrial depolarization could lead to PINK1 accumulation in the mitochondrial outer membrane and Parkin translocation to mitochondria, inhibiting mitochondrial fusion and trafficking and inducing mitophagy. This explanation could be supported by our finding that mitochondria in WFS1-deficient neurons were slightly depolarised. Another explanation would be that overactivation of the PINK1-Parkin pathway occurs independently of mitochondrial membrane potential, leading to the removal of healthy and polarised mitochondria. We earlier observed a similar phenomenon in mutant alpha-synuclein expressing neurons where PINK1-Parkin-dependent mitophagy started to eliminate polarised mitochondria [25]. It cannot be excluded that both of these explanations are also valid for WFS1-deficient neurons. Slight mitochondrial depolarisation may increase the rate of mitochondrial removal, and PINK1-Parkin dependent mitophagy could start to eliminate functional or at least partly functional mitochondria. This excessive mitochondrial removal should then lead to decreased mitochondrial density and ATP production, both of which were observed in WFS1-deficient neurons and compromise the bioenergetic status of cells.

Compared with the majority of other cell types, in which mitochondrial turnover is high, mitochondrial turnover is relatively low in neurons. It might therefore be suggested that neurons cannot afford to lose mitochondria at a high rate, as it would lead to energy deficits. Instead, it might be energetically more favourable for neurons to keep “partially defective” mitochondria than to consume them through mitophagy; in other words, “partially defective” mitochondria are the lesser evil. In contrast, under pathological conditions associated with increased levels of autophagy and mitophagy, excessive and unwanted mitochondrial clearance would lead to bioenergetic deficits harmful to neurons (in our case, an increased number of partially defective mitochondria and increased removal of these partially defective mitochondria, leading to reduced mitochondrial mass). It is likely that this event is not limited to *Wfs1*-deficient neurons but might also be observed in the future in other neurodegenerative conditions.

There is also some limited evidence in the literature that WFS1 is associated with Parkinson pathways. Shadrina et al. [26] demonstrated that the synonymous polymorphism C1645T in the *WFS1* gene increases the risk of Parkinson's disease in Russian patients. Kõks et al. [27] demonstrated recently that *WFS1* silencing in HEK cells primarily affected the expression of genes belonging to the Parkinson’s signalling ingenuity canonical pathway. Moreover, WFS1-deficient mice demonstrate impaired function of the dopaminergic system [28].

Another important discovery is that WFS1 deficiency delays neuronal development and impairs neuronal survival in primary neuronal culture. Ex vivo magnetic resonance imaging of the brains of *Wfs1*^-/-^ mice also demonstrated clear atrophy and/or degeneration of the brain stem, which is the main structure atrophied in Wolfram syndrome patients and the cause of death due to respiratory failure [1]. This is also consistent with an earlier clinical study suggesting that WFS1 had a pronounced impact on early brain development [2]. Compared with healthy and type 1 diabetic control groups, a cohort of young WS patients at relatively early stages of disease showed smaller intracranial volumes and preferentially affected grey matter volume and white matter microstructural integrity. According to our data, the link between WFS1 deficiency and delayed neuronal development appears to be mediated by impaired mitochondrial dynamics, because suppression of the PINK1-Parkin pathway also corrected the development delay. Our results do not allow us to elucidate the exact mechanism by which disturbed mitochondrial dynamics delays neuronal development; however, some putative mechanisms could be proposed. Impaired mitochondrial trafficking in WFS1-deficient neurons might negatively affect the delivery of energy-producing mitochondria to the sites where energy is most needed. For example, inhibition of mitochondrial transport results in the loss of mitochondria from peripheral nerve terminals that may reduce local ATP supply and affect ATP-dependent processes. Besides, any serious disturbances in mitochondrial fusion–fission dynamics may impair the maintenance of mitochondrial function and further compromise neuronal energy requirements. Disruption of mitochondrial fusion results in mitochondrial dysfunction and loss of respiratory capacity (for review, see [29]). Finally, excessive mitophagy that decreases mitochondrial mass will also affect the capacity of total energy production in neurons. Notably, neuronal growth is associated with increased energy demand and may be slowed by energy deficits.

Our discovery that WFS1 deficiency-elicited perturbations in Ca^2+^ homeostasis leads to disturbed mitochondrial dynamics and impaired neuronal development may help us to understand the pathophysiology of some psychiatric disorders. Indeed, WS has been associated with psychiatric pathologies (for review, see [30]), such as severe depression, psychosis, dementia, impulsive-aggressive behaviour leading to suicide attempts, and frequent hospitalization [31]. Heterozygous carriers of mutant *WFS1*, who are estimated to be as high as 1% of the general population, may also be at increased risk for mood disorders. Swift et al. [32] suggested that heterozygous WS carriers are 26-fold more likely to require psychiatric hospitalization compared with non-carriers, and these heterozygotes may constitute approximately 25% of all individuals hospitalized with depression and suicide attempts. These findings were confirmed in several further papers [33–35], although some reports failed to find an association [36–38]. This discrepancy is likely related to differences in cohorts of patients and requires further investigation. However, in general, our data are consistent with a growing body of evidence suggesting that impaired mitochondrial function (including mitochondrial dynamics) may lead to a disruption of normal neural plasticity and reduced cellular resilience, which may, in turn, promote the development of mood and psychotic disorders.

In conclusion, our data suggest a causal relationship between ER stress, cytosolic Ca^2+^ disturbances, impaired mitochondrial dynamics, and delayed neuronal development in WFS1-deficient neurons. This mechanism sheds new light on the development of neuronal abnormalities in Wolfram syndrome and points out potential therapeutic targets. Moreover, our results unravel two rather unexpected links having impact beyond the relatively rare Wolfram syndrome. Firstly, relatively mild ER stress/impaired ER Ca^2+^ release could seriously disturb mitochondrial dynamics, thus providing an explanation as to why alterations at the ER level could lead to a mitochondrial phenotype. Secondly, impaired mitochondrial dynamics could affect neuronal development, suggesting that proper mitochondrial dynamics might be crucial for neurodevelopment. Since alterations in WFS1 function seem to take place in different neurological disorders [30,32], our work may also have rather broad implications for understanding the role of mitochondrial dynamics in neuropsychiatric diseases.

## Methods

### Plasmids and Chemicals

Plasmids expressing scrambled shRNA or shRNA against rat *Wfs1* (KR46208N), rat *Atf6* (KR51427H), rat *Atf4* (R42749N), rat *Parkin* (KR50238N), and rat *Pink1* (KR55105N) were from SABiosciences. shRNAs against *Parkin* and *PINK1* have been validated by us earlier [20]. shRNAs against *ATF6* and *ATF4* supressed the expression of respective mRNAs by 74% and 68%. Plasmids expressing mitochondrial DsRed2 (632421) and EGFP (6085–1) were from Clontech. Mito-Keima was from Amalgaam (AM-V0251), and mito-KikGR1 was constructed as described earlier [39]. ATeam (51958), ATF6 (11975), ATF6-GL3 (11976), ATF4 (26114), ATF4-luc (21850), WFS1 wt (13011), WFS1 P724L (13012), IRE1α (13009), D1ER (36325), D3cpv (36323), DRP1 K38A, EGFP-LC3 (24920), HSPA5 wt (27164), HSPA5 T37G (27165), HSPA5 P495L (27166), NRF2 (21555), ORAI1 (21638), PSD-95 (15463), and pAAV-hSyn-DsRedExpress (22907) were obtained from Addgene (Cambridge, MA). The IP_3_R1 channel fragment (MmCD00312368) was from PlasmID, pRL-CMV (E2261) was from Promega Co., and mKate2-mito (FP187) was from Evrogen. XBP1ΔDBD-LUC was a kind gift from Dr. T. Iwawaki, YFP-Parkin from Dr. R. Youle, cytosolic aequorin from Dr. R. Rizzuto, DRP1 K38A from Dr. G. Szabadkai, pGL3-rNQO1 Dr. J. Alam, and PINK1 from Dr. E. Deas. DHPG (0805) and Bay K 8644 (1544) were from Tocris Bioscience, Brefeldin A (B6542) and Cyclopiazonic acid (C1530) were from Sigma-Aldrich, and Araguspongin B was from Cayman Chemical (10006797). All fluorescence dyes and culture media were from Life Technologies.

### Cell Cultures

Primary rat neuronal cultures were prepared from less than 1-d-old neonatal Wistar rats as described earlier [39]. Briefly, cortices were dissected in ice-cold Krebs–Ringer solution containing 0.3% BSA and then trypsinised in 0.8% trypsin for 10 min at 37°C. The cells were then triturated in a 0.008% DNase solution containing 0.05% soybean trypsin inhibitor. Cells were resuspended in Basal Medium Eagle with Earle's salts (BME) containing 10% heat-inactivated fetal bovine serum (FBS), 25 mM KCl, 2 mM glutamine, and 100 μg/ml gentamicin, and then plated onto 35-mm glass-bottomed dishes (MatTek, MA), which were pre-coated with poly-L-lysine, at a density of 10^6^ cells per dish in 2 ml of cell suspension. After incubating for 3 h, the medium was changed to Neurobasal-A medium containing B-27 supplement, 2 mM GlutaMAX-I, and 100 μg/ml gentamicin.

To prepare primary cultures of cerebellar granule cells, the cerebella from 8-d-old Wistar rats were dissociated by trypsinising in 0.25% trypsin at 35°C for 15 min, followed by trituration in a 0.004% DNase solution containing 0.05% soybean trypsin inhibitor. Cells were resuspended in BME containing 10% FBS, 25 mM KCl, 2 mM glutamine, and 100 μg/ml gentamicin. Neurons were plated onto 35 mm glass-bottomed dishes that were pre-coated with poly-L-lysine at a density of 1.3 × 10^6^ cells/ml. 10 μM cytosine arabinoside was added 24 h after plating to prevent the proliferation of glial cells.

Primary cortical neurons were isolated using the same protocol from 1-d-old wt and *Wfs1*-deficient mice [40] obtained from mating of background-matched wt and *Wfs1*-deficient mice, respectively. The permissions for the animal studies were given to E. Vasar (No. 39 and 29) and D. Safiulina (No. 51) by the Estonian National Board of Animal Experiments in accordance with the European Communities Directive of 24 November 1986 (86/609/EEC).

PC12 or PC6 cells were grown in RPMI-1640 medium supplemented with 10% horse serum and 5% FBS on collagen IV-coated 100-mm plastic dishes or on 35-mm glass-bottomed dishes. All culture media and supplements were obtained from Invitrogen.

### Transfection

Neurons were transfected at DIV2 (with exception of neuronal maturation and axonal growth experiments). For transfection of cells growing on glass-bottomed dishes, the conditioned medium was replaced with 100 μl Opti-MEM I medium containing 2% Lipofectamine 2000 and 1 to 2 μg of total DNA with an equal amount of each different plasmid. The dishes were incubated for 3 to 4 h, after which fresh medium was added. For biochemical analyses, the cells were transfected in 100-mm plastic dishes as described above, except that the total volume of the transfection mixture was increased with proportionally adjusted Lipofectamine 2000 and DNA. For some experiments with shRNA-expressing plasmids that also contained a neomycin resistance gene (shRNA efficiency testing and Parkin translocation), the PC12 cell medium was supplemented with 200 μg/ml G418 for 6–7 d.

Note that despite relatively low transfection efficiency in neurons, the transfected plasmids were mostly localised to the same cells. When neurons were transfected with plasmids encoding mitochondrial CFP, mitochondrial YFP, and mitochondrial mKate2, 93 ± 1% transfected cells expressed all three markers, 5 ± 1% expressed two markers, and 2 ± 0.4% expressed one marker (*n* = 4 dishes; 100 cells were analysed per dish; S17 Fig).

### Mitochondrial Fusion Rate Analysis

For mitochondrial fusion rate analysis, cortical neuronal cultures transfected with mito-KikGR1 plasmid and plasmids of interest as described earlier [39] and examined at DIV 7–8. A laser scanning confocal microscope (LSM 510 Duo, Carl Zeiss Microscopy GmbH) equipped with a LCI Plan-Neofluar 63×/1.3 water immersion DIC M27 objective was used. The temperature was maintained at 37°C using a climate chamber. For fusion acquisition, mito-KikGR1 was illuminated with a 488-nm argon laser line to visualize the intense green mitochondrial staining. Selected mitochondria were then photoconverted to red using a 405-nm diode laser and illuminated using a 561 nm DPSS laser. The images were taken at 10-s intervals for 10 min, the fate of all activated mitochondria was followed throughout the time-lapse, and the fusion and fission events were recorded.

### Mitochondrial Density and Length

For whole-cell mitochondrial density measurements, the neurons were transfected with GFP, mitochondrial pDsRed2, scrambled shRNA or shRNA, and plasmids of interest. At DIV 8, the entire axon and dendrites from randomly selected neurons were visualised using a laser scanning confocal microscope. Neurons were reconstructed using Neurolucida and LSM5 software, and mitochondrial density was analysed. Mitochondrial length measurements were performed as described previously [39].

### Mitophagy Assays

Primary cortical neurons transfected with mitochondrially targeted Keima plus scrambled shRNA, and plasmids of interest were studied at DIV 7–8. The excitation spectrum of Keima shifts from 440 to 586 nm when mitochondria are delivered to acidic lysosomes, which enables quantification of mitophagy. Images were acquired by a laser scanning confocal microscope using the laser lines 458 nm (green, mitochondria at neutral pH) and 561 nm (red, mitochondria under acidic pH) at 5–6 d after transfection, and red dots were counted blindly.

In another set of experiments, neurons were transfected with pEGFP-LC3, mKate2-mito, and scrambled shRNA or Wfs1 shRNA. The co-localization of EGFP-LC3 dots and mitochondrial mKate2-mito was analysed.

### Cytosolic ATP Measurement

Neurons expressing the ATP sensor ATeam and scrambled or *Wfs1* shRNA were excited using a 458 nm line (10%) of Ar-laser, the CFP emission was acquired at 465–500 nm and the FRET signal at 520–570 nm. The ratio of the FRET/CFP fluorescence intensity was calculated from the signal coming from the cytosol.

### Mitochondrial Membrane Potential

For membrane potential measurements, primary neurons (plated at lower density, 2.5 x 10^5^ cells/ml) were transfected with 20 nM validated siRNA against *Wfs1* (Sigma-Aldrich: SASI_Rn02_00265296 Rat NM_031823; *Wfs1* siRNA suppressed 80 ± 1% of endogenous WFS1 expression in primary cortical cells as estimated by RT-PCR, *n* = 3) using the N-TER Nanoparticle siRNA Transfection System (Sigma-Aldrich). Briefly, a mixture of target and scrambled siRNA (20 nM) diluted in siRNA buffer and NTER transfection reagent diluted in ddH_2_0 was preincubated at RT for 20 min. Growth medium was then removed and replaced with Opti-MEM I containing the target or scrambled siRNA mixture. After a 3-h incubation at 37°C, Opti-MEM I was changed to Neurobasal-A medium containing B-27 supplement, 2 mM GlutaMAX-I, and 100 μg/ml gentamicin. After transfection, the cells were incubated for 72–96 h in a humidified 5% CO2/95% air incubator at 37°C. The N-TER Nanoparticle siRNA Transfection System was relatively non-toxic, yielding a survival rate of 82 ± 4% (five dishes and five dishes in vehicle treated group) at 16 h after transfection (estimated with LIVE/DEAD Viability/Cytotoxicity Kit, for mammalian cells [Invitrogen]).

For JC-10 loading, siRNA-transfected dishes were kept in 10 μM JC-10 dissolved in culture media and incubated at 37°C for 20 min. After dye-loading, the cells were kept in Krebs-Ringer solution supplemented with 1mM Ca^2+^ and visualized using a laser scanning confocal microscope equipped with a LCI Plan-Neofluar 63×/1.3 water immersion DIC M27 objective. Dishes were then treated with 5 μM FCCP to obtain background values.

For visualizing mitochondria with preserved membrane potential in autophagosomes, the neurons were transfected with LC3-EGFP and *Wfs1* shRNA. On the fourth day after transfection, the cells were stained with the mitochondrial membrane potential sensitive dye tetramethylrhodamine ethyl ester (TMRE) at a concentration of 50 nM in complete NeurobasalTM-B medium at 37°C for 30 min and visualized in Krebs-Ringer solution supplemented with 1 mM Ca^2+^ using confocal microscope equipped with a 100×/oil objective.

### Calcium Measurements

For Fluo-4 based measurements, neurons were transfected with scrambled shRNA or Wfs1 shRNA, plasmids of interests, and mKate2-mito to visualise transfected cells. Five days later, cells were loaded with 5 μM Fluo-4 AM in Hank’s Balanced Salt Solution (HBSS with Ca^2+^ and Mg^2+^) for 30 min at 37°C, followed by 10 min incubation in HBSS without dye to allow complete de-esterification of intracellular AM esters. Fluo-4 AM was excited using a 488-nm argon laser, and emitted fluorescence was quantified using a LSM 510 confocal microscope. Time-lapse images were recorded at 2-s intervals for 1 min before and 2 min after the induction of Ca^2+^ transients. Cytosolic Ca^2+^ transients were induced by 25 mM KCl, 200 μM DHPG, 2 mM glutamate, or by the photoactivatable membrane-permeant caged derivative of IP_3_. In the latter case, cells were co-loaded with 2.5 μM Fluo4-AM and 4 μM Ins(1,4,5)P_3_-PM (SiChem) for 90 min at 37°C. Photolysis of caged IP_3_ by irradiating the individual cells with near-UV laser (405 nm) for 10 s was used to release active IP_3_. Fluorescence signals from single transfected neurons identified with mKate2-mito were analysed, and mean changes in fluorescence intensities were calculated. Examples of Fluo-4 time-lapse images in control or *Wfs1*-siRNA transfected neurons are depicted in S18 Fig.

For the FRET-based analysis of cytosolic and ER Ca^2+^ levels, neurons were transfected with the genetically encoded FRET-based chameleon indicators D3cpv or D1ER, respectively, and scrambled shRNA or Wfs1 shRNA. Transfected neurons were rinsed once and then allowed to equilibrate in HBSS containing Ca^2+^ and Mg^2+^ for 20 min. Neurons were excited at 405 nm and emission acquired at 465–510 (CFP) and 520–555 (FRET).

For bioluminescence-based calcium measurements, neurons were transfected with cytosolic aequorin together with shRNAs and plasmids of interest. Five days later, cells were incubated for 30 min with 3 μM ViviRen Live Cell Substrate (Promega) in Krebs buffer containing 1 mM CaCl_2_ and 0.5% BSA at 37°C. Ca^2+^ uptake was stimulated by 25 mM KCl and experiments were terminated by lysing the cells with 2.5% Triton X-100 in Ca^2+^-rich solution to measure the maximal activity of aequorin. Aequorin luminescence was monitored by Victor X5 Multilabel Plate Reader (PerkinElmer).

### Luciferase Reporter Assays

Primary neurons or PC12 cells growing in 96-well plates were transfected with the desired firefly luciferase reporter plasmid, *Renilla* luciferase, and plasmids of interest. The luciferase assays were performed 48–96 h later using Dual-Glo Luciferase Assay reagent (Promega) according to the manufacturer's instructions. The promoter activities for NRF2, ATF6, ATF4, and IRE firefly luciferase luminescence were normalized to *Renilla* luciferase signal.

### Neuronal Maturation, Axonal Growth, and Synaptic Density

For neuronal maturation experiments, cortical neurons were transfected at DIV1 with a plasmid expressing neuron-specific pAAV-hSyn-DsRed1 and scrambled shRNA or Wfs1 shRNA. Live-cell morphology was visually examined using a fluorescence microscope (Olympus IX70, 20x/0.70 water immersion objective) on randomly selected fields (minimum 30 fields per group) on the indicated days in culture. Neurons were classified into the four subgroups depending on their maturation stage (Type I: lamellipodia; Type II: immature neuron, sprouting of several minor neurites; Type III: axon and dendrite formation, neuronal polarisation and branching; Type IV: neuron with adult-like morphology, ongoing maturation of differentiated processes).

For the axonal growth analysis, images of cultured cortical neurons (DIV2 to DIV6) were captured using an Olympus IX70 inverted microscope with a 20x objective and traced manually using Neurolucida software (MBF Bioscience). The length of the axonal tree, length of the longest axon, and number of axon tips were measured using Neurolucida Explorer.

For synapse detection, neurons were transfected at DIV2 with GFP and shRNAs. Neurons were fixed and permeabilized at DIV4, DIV6, or DIV18 using the Image-iT Fixation/Permeabilization Kit (Life Technologies) according to the manufacturer’s protocol. Fixed neurons were then incubated with the primary antibody mouse anti-PSD95 (1:1000, ab2723, Abcam) in the presence of 3% normal goat serum at 4°C for 24 h. After washing, the cells were further incubated with the secondary antibody goat anti-mouse DyLight 594 (1:1000, ab96873, Abcam) at room temperature for 1 h and visualised using LSM510 confocal microscope (63×/1.3 water immersion objective). The immunofluorescent puncta close to soma colocalizing with GFP were quantified manually. The PSD-95 positive puncta co-localizing with GFP marked neurites were quantified manually.

### Magnetic Resonance Imaging

The majority of mice were generated from Wfs^+/-^ male and female [40] breeding pairs. Additional Wfs^+/+^ mice (2) were generated from a separate breeding pair on a similar background. Mice were housed in a temperature- and humidity-controlled room. Food and water were available ad libitum, and mice were kept on a 12:12 h light:dark cycle. At 1 y of age, mice were deeply anesthetized and perfused with 0.1 M PBS followed by 4% paraformaldehyde (4°C). Brains were left in skulls to preserve anatomy and incubated in 4% PFA overnight at 4°C and then in PBS until 2 days prior to imaging. Skulls were then placed in 2 mM gadovist in PBS and incubated at 4°C with rocking until imaging. A T2 RARE sequence was used for imaging using a 94/20 Bruker BioSpec small animal MRI with the following parameters: Tr, 900 ms; TE, 47.13 ms; imaging matrix, 512 x 360 x 80; spatial resolution, 0.0444 x 0.03 x 0.2 mm for an imaging time of approximately 3 h and 4 min. Volumes were segmented manually by an observer blinded to genotype using ITK-SNAP (V3.2.0). For the cortex at the level of the striatum, the volume of cortex from bregma +1.70 mm to -2.18 mm was quantified. For the optic nerve, volumes were calculated for optic nerve rostral to the optic chiasm. For brain stem (pons and medulla), the most rostral portions of the pons and the most caudal portion of the medulla ventral to the cerebellum are not included in the Paxinos atlas; thus, quantification of brain stem began at the most rostral portion of the pons, ventral to the interpeduncular nucleus and dorsal to the mammillary body (approximately bregma +3.62 mm), and ended at the termination of the overlying cerebellum (approximately bregma -8.5 mm).

### RT-PCR

Total RNA was isolated from age-matched *Wfs1*^-/-^ and *Wfs1*^+/+^ using the Qiagen RNeasy Mini Kit. Conversion to cDNA was performed by reverse transcription using 1 μg of total RNA with SuperScript III RT kit (Invitrogen). Specific primers were designed for amplification of Mfn2, Mfn1, Opa1, Drp1, Fis1, Miro2, and Cox4 genes. qPCR was performed on an ABI PRISM 7900HT Sequence Detection System. Reactions were performed using an ABI SYBRGreen PCR Master Mix, and raw data were analysed using the ΔΔCt method. All Ct values were normalised to the control gene synaptophysin (SYN).

### Statistics

Data are presented as the mean ± SEM. The number of replicates for each type of experiments is given in S2 Table. The D’Agostino-Pearson omnibus test was used to test the normality of distribution. *t* tests, Mann Whitney test, Wilcoxon signed rank test, one-way ANOVAs followed by Bonferroni posthoc test (selected pairs), or Kruskal-Wallis tests followed by the Dunn test were used to compare differences between experimental samples and control groups. Two-way ANOVAs were used to analyse interactions between two treatments. *P* values of less than 0.05 were considered statistically significant.

## Supporting Information

S1 Data Excel spreadsheet containing, in separate sheets, the underlying numerical data and statistical analysis for figure panels 1B–1E, 1G, 1H, 1J, 1K, 2A–2D, 3A–3L, 4A–4I, 5A–5L, 6B, 6D–6G, 6I, 7E–7H, 8B–8K, 8M, 8N, 8P, 8Q, S1B, S2, S4B, S6, S8B, S9A–S9D, S10A, S10B, S11, S12, S13A, S13B, S15A, S15B, S16A–S16D, S18B, and S18E. (XLSX)

S1 Fig *Wfs1* shRNA suppresses endogenous WFS1 expression in PC12 cells. (A) For scrambled shRNA, *Wfs1* shRNA, and WFS1 overexpression, immunoreactive bands of WFS1 and GAPDH were visualised using an Odyssey Infrared Imaging System. (B) The signal intensities of WFS1 were normalized twice—to those of GAPDH in the same samples and to the transfection efficiency. Results are taken from two independent experiments and include five samples per group. ***p < 0.001 compared with the control group. Underlying data is shown in S1 Data.
(TIF)

S2 Fig mRNA expression of the main fusion and fission proteins in *Wfs1*^+/+^ and *Wfs1*^-/-^ brains. The mRNA levels of MFN2, MFN1, OPA1, DRP1, FIS1, MIRO2, and COX4 were normalised to synaptophysin mRNA levels and expressed relative to the *Wfs1*^**+/+**^ group (*n* = 4–6 brains per group). Underlying data is shown in S1 Data.
(TIF)

S3 Fig *Wfs1* silencing increases the number of mitochondria colocalizing with an autophagosome marker. Neurons were transfected with the autophagosome marker EGFP-LC3, the mitochondrial marker mKate2-mito, and scrambled shRNA or *Wfs1* shRNA, and the number of co-localizations was analysed 4 d later.
(TIF)

S4 Fig *Wfs1* silencing enhances LC3-I conversion to LC3-II. PC6 cells were transfected with EGFP-LC3 and scrambled shRNA or *Wfs1* shRNA or wt WFS1 and selected using G418 for 7 d, after which total cell lysates were prepared. (A) Western blot showing decreased levels of monomeric WFS1. The magnified inset shows the increased LC3-I conversion to LC3-II in the Wfs1 shRNA-transfected group. The latter was partially rescued by overexpressing wt shRNA-insensitive WFS1. Note the increased expression of heteromeric WFS1 in this group. (B) Densitometric band-intensity ratio of LC3-I/LC3-II based on three samples in each group. **p < 0.01 compared with the control group and ^#^*p* < 0.05 compared with the *Wfs1* shRNA group. Underlying data is shown in S1 Data.
(TIF)

S5 Fig Kymograms showing the movement of mitochondria in control and *Wfs1* shRNA-silenced neurons expressing mitochondrial Kikume-Green. The upper panels show the first frame of the time course, and the lower panels show the movement of mitochondria over the following 10 min.
(TIF)

S6 Fig WFS1 deficiency decreases the mitochondrial contact rate. The reduced contact rate (number of contacts per mitochondrion per minute) in cortical neurons isolated from *Wfs1*^-/-^ mice is restored by overexpression of wt WFS1. *Wfs1* silencing by *Wfs1* shRNA in *Wfs1*^+/+^ neurons has the same effect on the contact rate as genetic *Wfs1* knock-out. * *p* < 0.05 and ** *p* < 0.01 versus indicated group. Underlying data is shown in S1 Data.
(TIF)

S7 Fig Examples of JC-10 staining in control or *Wfs1*-siRNA transfected neurons. The green channel shows monomeric JC-10, and the red channel demonstrates aggregated JC-10.
(TIF)

S8 Fig The number of TMRE-positive mitochondria surrounded by an EGFP-LC3-positive autophagosome was increased in *Wfs1*-silenced neurons. (A) Example of a TMRE-positive mitochondrion surrounded by an EGFP-LC3-positive autophagosome (indicated by arrow) in a *Wfs1*-shRNA transfected neuron. (B) Quantification of autophagosomes colocalising with TMRE per optical slice in the neuronal body. **p < 0.01 compared with control group. Underlying data is shown in S1 Data.
(TIF)

S9 Fig Calcium release and uptake properties of ER in primary cortical neurons. (A,B) Neurons transfected with scrambled shRNA or *Wfs1* shRNA were permeabilised, loaded with the intra-reticular Ca^2+^ sensor Mag-fluo-4, and incubated with 100 nM Ca^2+^. IP_3_ (100 nM) induces much stronger ER Ca^2+^ release than caffeine (5 mM) (A). Maximal values of Ca^2+^-dependent ER fluorescence after incubation with 100 nM Ca^2+^ (B). (C–D) Cytosolic Ca^2+^ transients (C) were elicited by 30 μM cyclopiazonic acid (CPA; blocking ER Ca^2+^ uptake) in neurons transfected with the FRET-based cytosolic Ca^2+^ sensor cytoD3cpv and scrambled shRNA or *Wfs1* shRNA. No difference was observed in the amplitude of Ca^2+^ transient (D).
(TIF)

S10 Fig *Wfs1* silencing had no effect on basal ER calcium. (A) Neurons were transfected with the ER-targeted second-generation Ca^2+^ sensor cameleon (D1ER) and treated with 30 μM CPA (blocking ER Ca^2+^ uptake, black line) or 200 μM DHPG (generating IP_3,_ blue line) to induce a decrease in ER Ca^2+^ content. This experiment was performed to demonstrate that the probe responds to treatments decreasing ER Ca^2+^ levels. (B) Neurons were transfected with D1ER and scrambled or *Wfs1* shRNAs. The results show no difference between control and WFS1-deficient neurons. Underlying data is shown in S1 Data.
(TIF)

S11 Fig Overexpression of IP_3_R does not supress *Wfs1* silencing-induced ER stress. Neurons were transfected with plasmids expressing an ATF6 firefly luciferase reporter, *Renilla* luciferase, and scrambled shRNA or *Wfs1* shRNA with or without IP_3_R. Indeed, overexpression of IP_3_R increases rather than supresses ATF6 reporter luminescence. *p < 0.05 and ***p < 0.001 versus indicated group. Underlying data is shown in S1 Data.
(TIF)

S12 Fig Overexpression of ORAI1 rescues impaired neuronal development in WFS1 deficiency. Neurons were transfected with the neuronal marker pAAV-hSyn-DsRed1 and scrambled shRNA or *Wfs1* shRNA with or without ORAI1 at DIV1. Overexpression of ORAI1 restores the percentage of mature neurons in WFS1-silenced neurons, estimated at DIV4. *p < 0.05 and **p < 0.01 compared with indicated groups. Two-way ANOVA, *p* = 0.031 for interaction. Underlying data is shown in S1 Data.
(TIF)

S13 Fig *Wfs1* silencing leads to a mild Parkin translocation to mitochondria. (A) PC12 cells were transfected with YFP-Parkin and scrambled shRNA, *Wfs1* shRNA, or wt PINK1 and visualised 6 d later. The fraction of cells with YFP-Parkin translocated to mitochondria is higher in the *Wfs1* shRNA-expressing group and in the PINK1-overexpressing group, used as a positive control. Underlying data is shown in S1 Data. (B) PC6 cells were transfected with YFP-Parkin and scrambled shRNA, *Wfs1* shRNA, or wt WFS1 and visualised 5 d later. The fraction of cells with YFP-Parkin translocated to mitochondria is higher in the *Wfs1* shRNA-expressing group. *p < 0.05 and **p < 0.01 compared with the scrambled shRNA group.
(TIF)

S14 Fig Examples of cortical neurons transfected with YFP-Parkin and scrambled shRNA, *Wfs1* shRNA, or wt WFS1. Notice the increased aggregation of YFP-parkin in *Wfs1* shRNA expressing neurons.
(TIF)

S15 Fig *PINK1*- or *Parkin*-silencing does not prevent WFS1 deficiency-induced ER stress and cytosolic Ca^2+^ disturbances. (A) Neurons were transfected with plasmids expressing ATF6 firefly luciferase reporter, *Renilla* luciferase, and scrambled shRNA or *Wfs1* shRNA. Additional co-transfection with *PINK1* shRNA or *Parkin* shRNA does not attenuate ATF6 activation induced by Wfs1 deficiency. (B) Neurons transfected with scrambled shRNA or *Wfs1* shRNA were co-loaded with cytosolic Ca^2+^ sensor Fluo-4. Transfected cells were visualised by co-transfection with the mitochondrial marker mKate2-mito. *PINK1* and *Parkin* silencing does not normalise the lowered amplitude of KCl-induced Ca^2+^ transients associated with WFS1 deficiency. **p < 0.01 and **p < 0.01 compared with indicated groups. Underlying data is shown in S1 Data.
(TIF)

S16 Fig Negative dominant (nd) DRP1 normalises mitochondrial dynamics and density as well as neuronal development in WFS1-deficient neurons. (A–C) Under conditions of WFS1 deficiency, nd DRP1 expression normalises mitochondrial fusion rate (A), mitochondrial density (B), and axon lengths (C). (D) nd DRP1 prevents Brefeldin-induced (5 μM for 48 h) inhibition of mitochondrial fusion rate. *p < 0.05 and **p < 0.01 compared with respective scrambled shRNA groups and ^#^*p* < 0.05, ^##^*p* < 0.01, and ^###^ *p* < 0.001 compared with respective *Wfs1* shRNA groups. Underlying data is shown in S1 Data.
(TIF)

S17 Fig Example images showing the co-localisation of co-transfected mitochondrial CFP, mitochondrial YFP, and mitochondrial mKate2 in primary cortical neurons. (TIF)

S18 Fig Examples of Fluo-4 time-lapse images in control or *Wfs1*-siRNA transfected neurons. (A–C) Neurons transfected with the mitochondrial marker mKate2-mito and scrambled shRNA were loaded with the Ca^2+^ sensor Fluo-4. (A) A single image of an mKate2-mito-positive neuron marked with a white box. (B) A time-lapse series of images of a transfected neuron (KCl was added at 63 s). (C) ROI intensity during this time-lapse. The ROI is visible in the first image of the time lapse series. (D–F) Neurons transfected with the mitochondrial marker mKate2-mito and *Wfs1* shRNA were loaded with the Ca^2+^ sensor Fluo-4. (D) A still image of an mKate2-mito-positive neuron marked by white box. (E) A time lapse series of images of a transfected neuron. (F) ROI intensity throughout the time-lapse. The ROI is visible in the first image of the time lapse series. Underlying data is shown in S1 Data.
(TIF)

S1 Table Number of neurons at different stages of development in control and *Wfs1-*shRNA transfected cultures at DIV2, 4, 6, and 8. (DOCX)

S2 Table Number of replicates used in experiments. (DOCX)

S1 Video Fusion event between photoactivated (red) and non-activated (green) mitochondria (bar 2 μm). (MP4)

S2 Video Mitochondrial trafficking in a scrambled shRNA-transfected neuron. (MP4)

S3 Video Mitochondrial trafficking in a *Wfs1* shRNA-transfected neuron. (MP4)

## Abbreviations

CaM: calmodulin
DHPG: dihydroxyphenylglycine
DIDMOAD: diabetes insipidus, diabetes mellitus, optical atrophy, and deafness
ER: endoplasmic reticulum
FRET: fluorescence resonance energy transfer
IP3R: inositol 1,4,5-trisphosphate receptor
ROS: reactive oxygen species
TMRE: tetramethylrhodamine ethyl ester
WFS1: Wolfram syndrome 1
WS: Wolfram syndrome
wt: wild-type
