# Supplementary figures and images for: Role of Mitochondrial Dynamics in Neuronal Development: Mechanism for Wolfram Syndrome
Source: PLoS Biol. 2016 Jul 19;14(7):e1002511. doi: 10.1371/journal.pbio.1002511 (PMC4951053; doi:10.1371/journal.pbio.1002511)

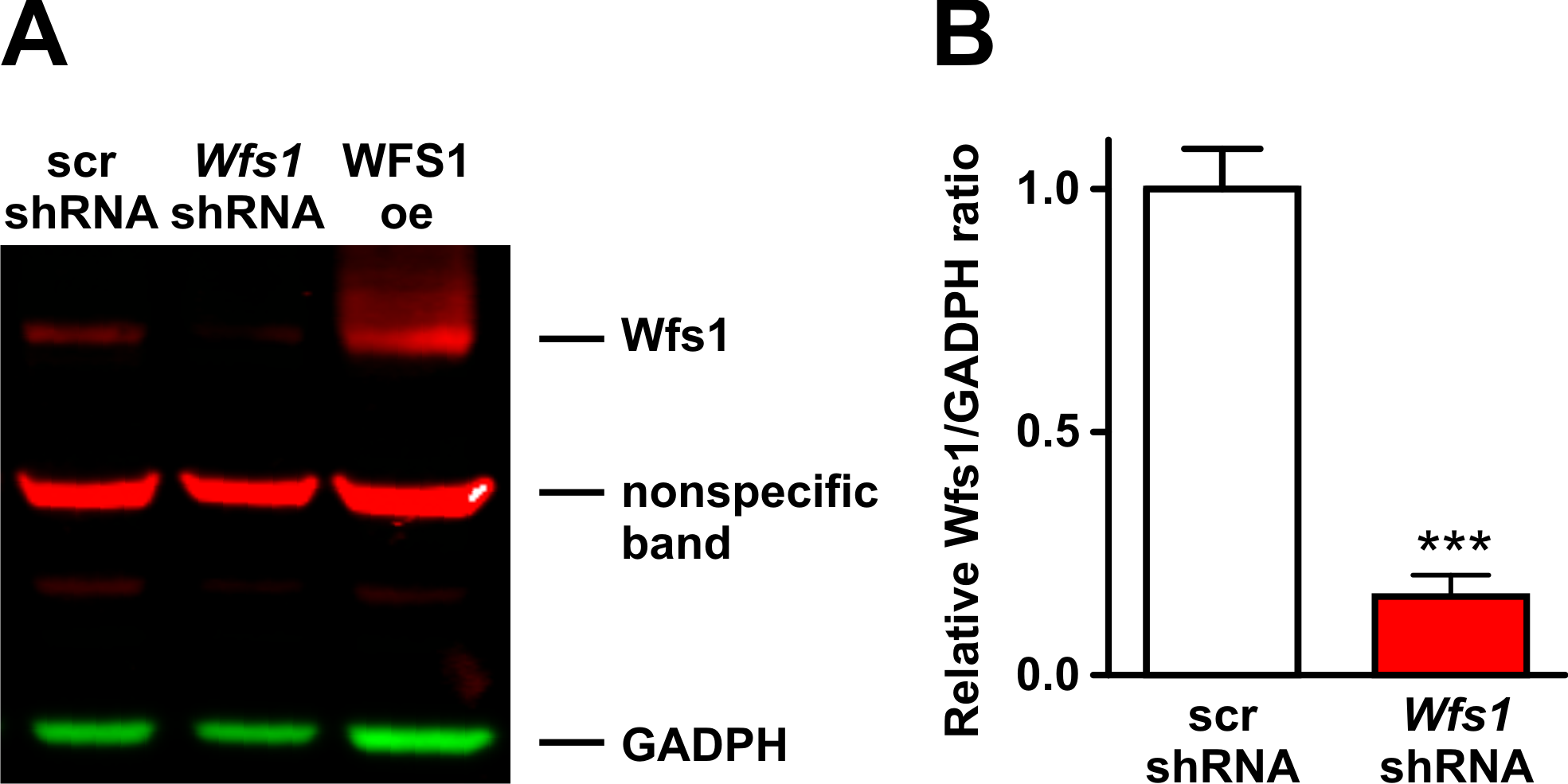

Supplement: S1 Fig — (A) For scrambled shRNA, Wfs1 shRNA, and WFS1 overexpression, immunoreactive bands of WFS1 and GAPDH were visualised using an Odyssey Infrared Imaging System. (B) The signal intensities of WFS1 were normalized twice—to those of GAPDH in the same samples and to the transfection efficiency. Results are taken from two independent experiments and include five samples per group. ***p < 0.001 compared with the control group. Underlying data is shown in S1 Data. (TIF) [file pbio.1002511.s002.tif]

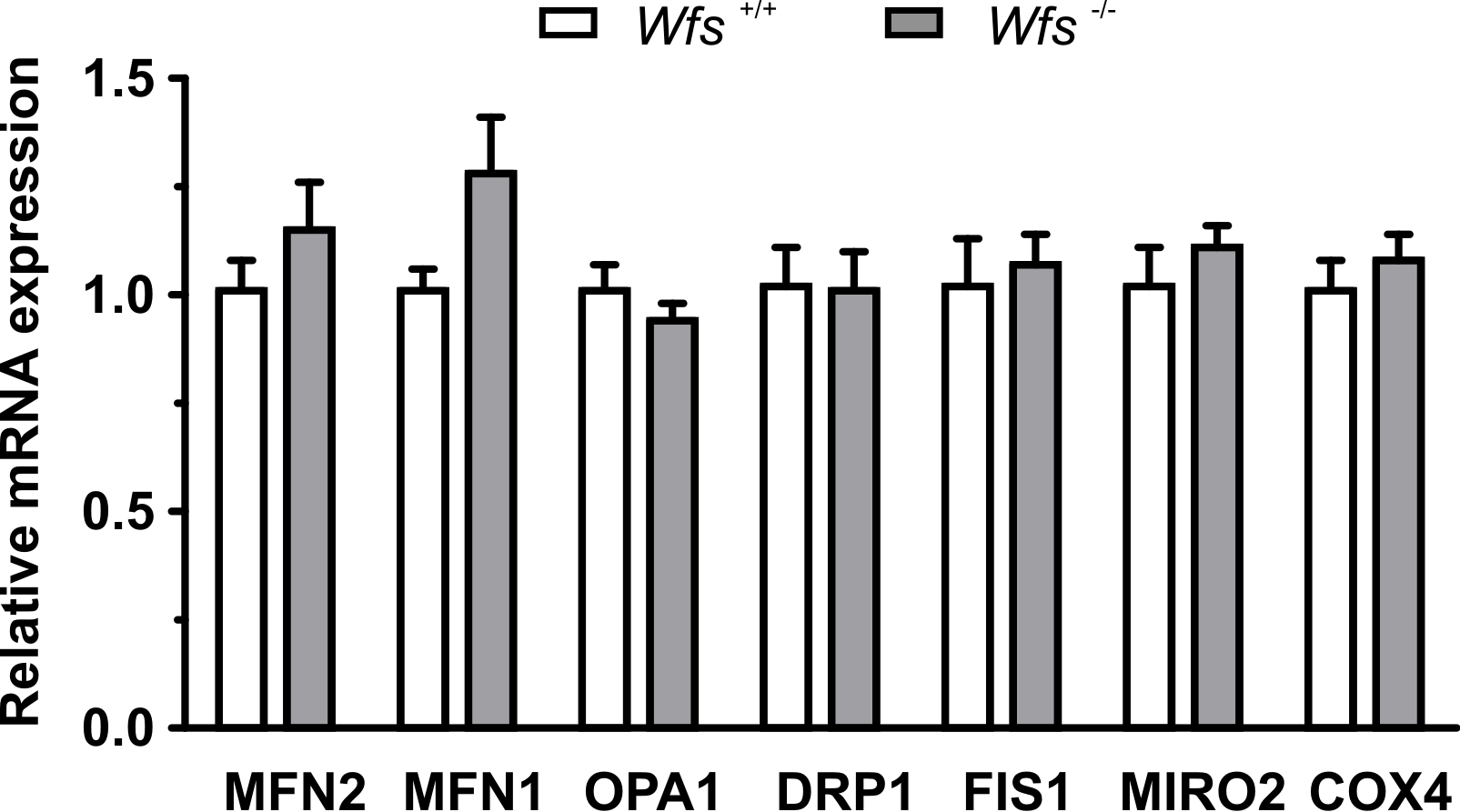

Supplement: S2 Fig — The mRNA levels of MFN2, MFN1, OPA1, DRP1, FIS1, MIRO2, and COX4 were normalised to synaptophysin mRNA levels and expressed relative to the Wfs1+/+ group (n = 4–6 brains per group). Underlying data is shown in S1 Data. (TIF) [file pbio.1002511.s003.tif]

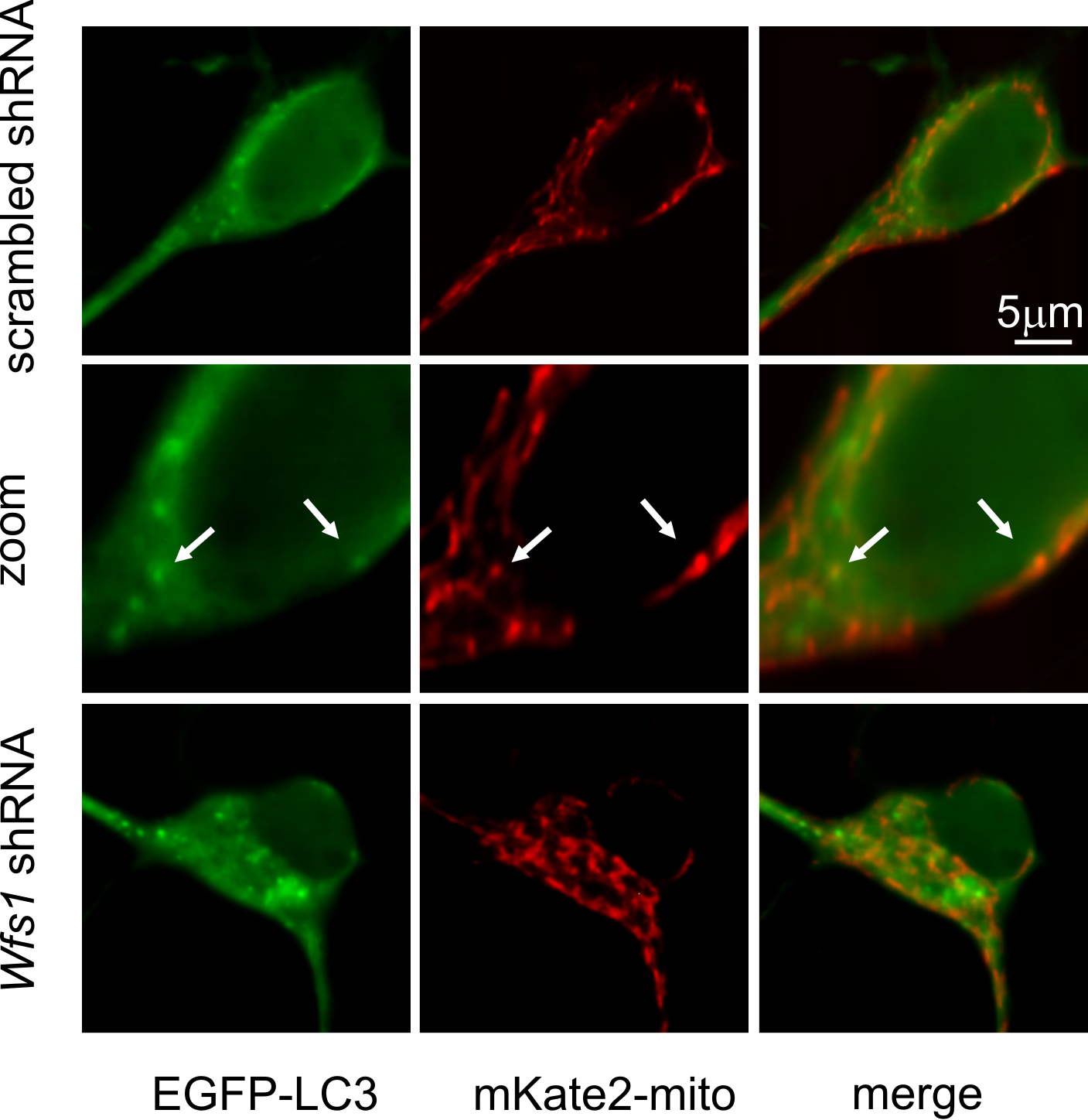

Supplement: S3 Fig — Neurons were transfected with the autophagosome marker EGFP-LC3, the mitochondrial marker mKate2-mito, and scrambled shRNA or Wfs1 shRNA, and the number of co-localizations was analysed 4 d later. (TIF) [file pbio.1002511.s004.tif]

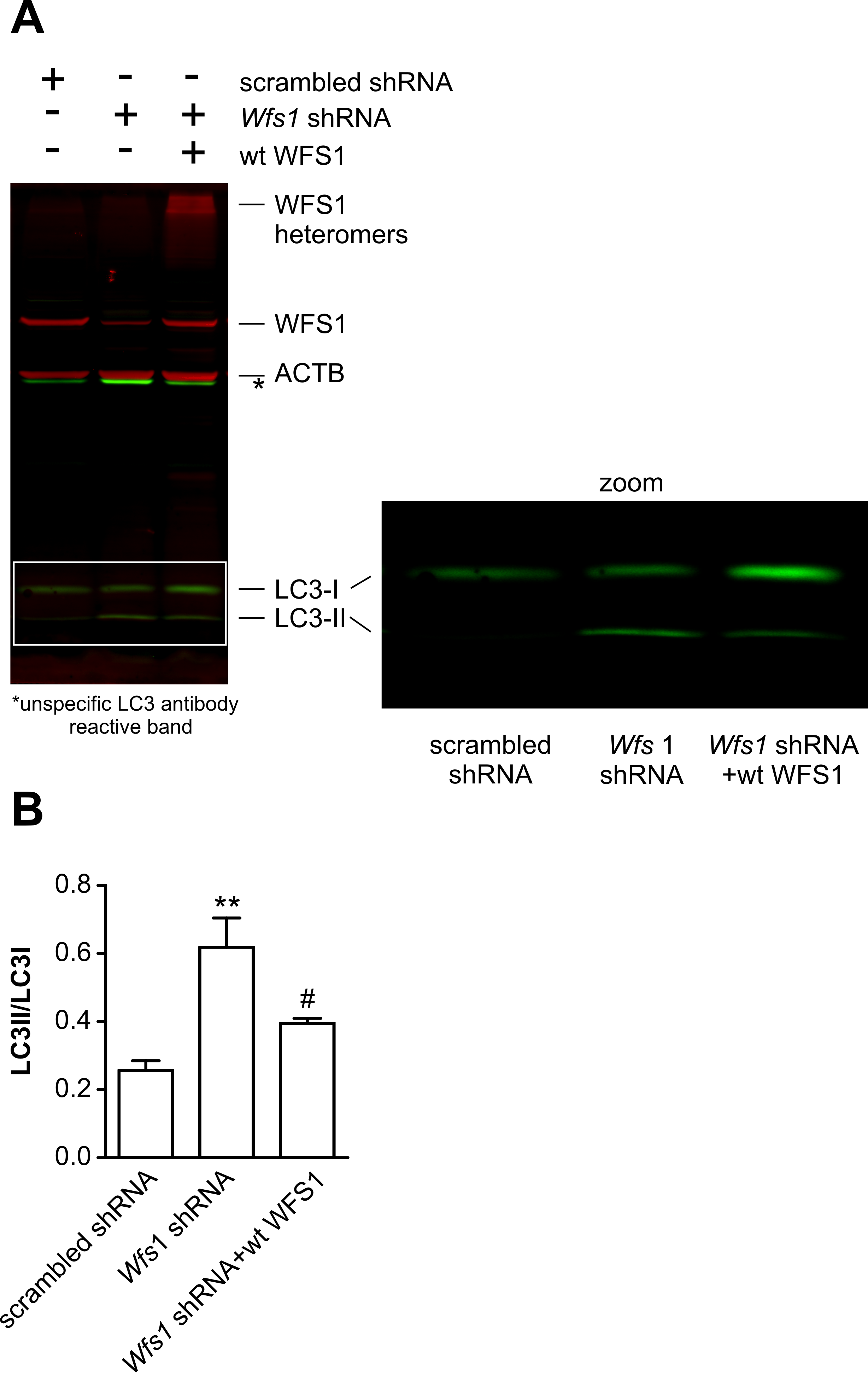

Supplement: S4 Fig — PC6 cells were transfected with EGFP-LC3 and scrambled shRNA or Wfs1 shRNA or wt WFS1 and selected using G418 for 7 d, after which total cell lysates were prepared. (A) Western blot showing decreased levels of monomeric WFS1. The magnified inset shows the increased LC3-I conversion to LC3-II in the Wfs1 shRNA-transfected group. The latter was partially rescued by overexpressing wt shRNA-insensitive WFS1. Note the increased expression of heteromeric WFS1 in this group. (B) Densitometric band-intensity ratio of LC3-I/LC3-II based on three samples in each group. **p < 0.01 compared with the control group and #p < 0.05 compared with the Wfs1 shRNA group. Underlying data is shown in S1 Data. (TIF) [file pbio.1002511.s005.tif]

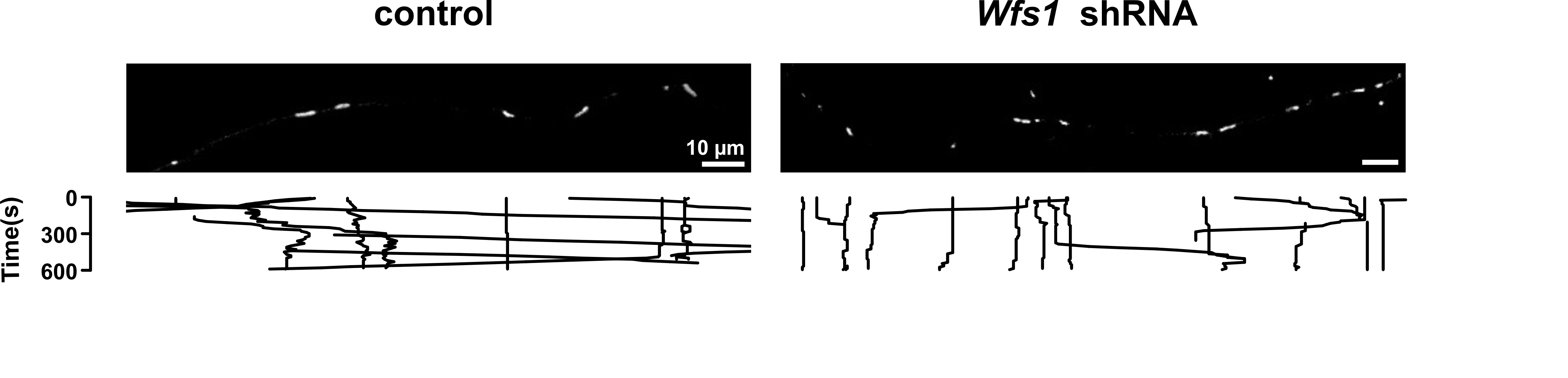

Supplement: S5 Fig — The upper panels show the first frame of the time course, and the lower panels show the movement of mitochondria over the following 10 min. (TIF) [file pbio.1002511.s006.tif]

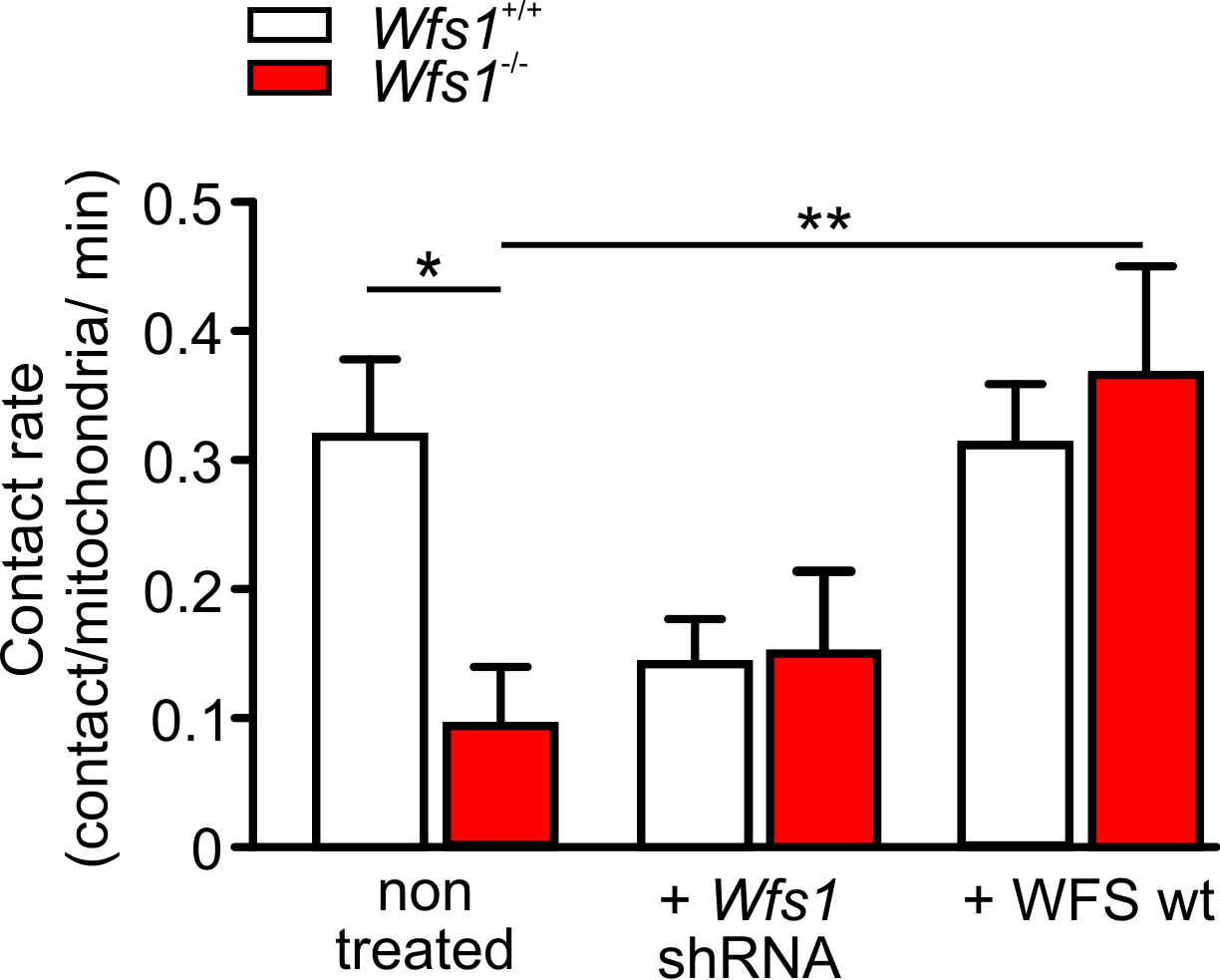

Supplement: S6 Fig — The reduced contact rate (number of contacts per mitochondrion per minute) in cortical neurons isolated from Wfs1-/- mice is restored by overexpression of wt WFS1. Wfs1 silencing by Wfs1 shRNA in Wfs1+/+ neurons has the same effect on the contact rate as genetic Wfs1 knock-out. * p < 0.05 and ** p < 0.01 versus indicated group. Underlying data is shown in S1 Data. (TIF) [file pbio.1002511.s007.tif]

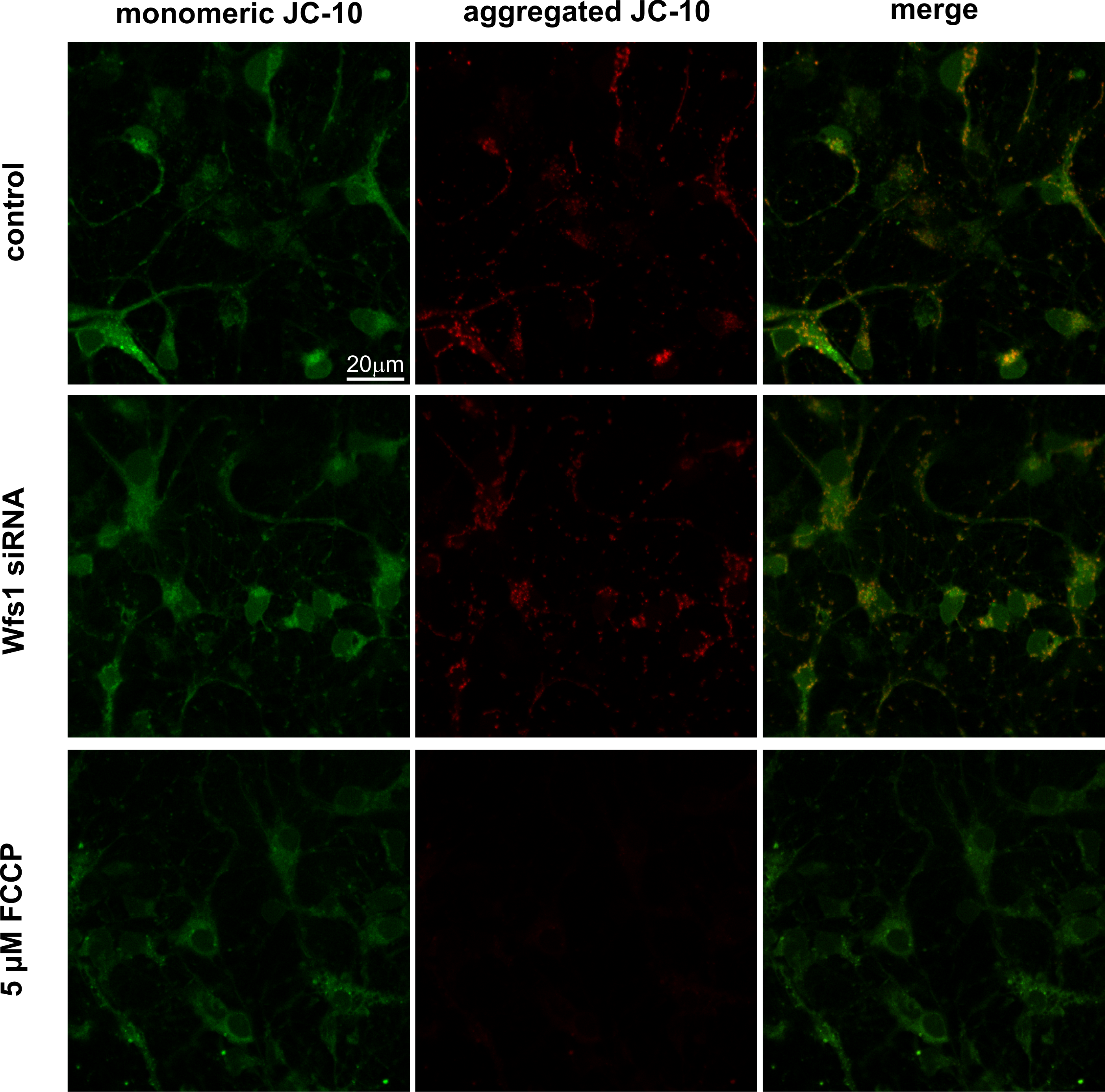

Supplement: S7 Fig — The green channel shows monomeric JC-10, and the red channel demonstrates aggregated JC-10. (TIF) [file pbio.1002511.s008.tif]

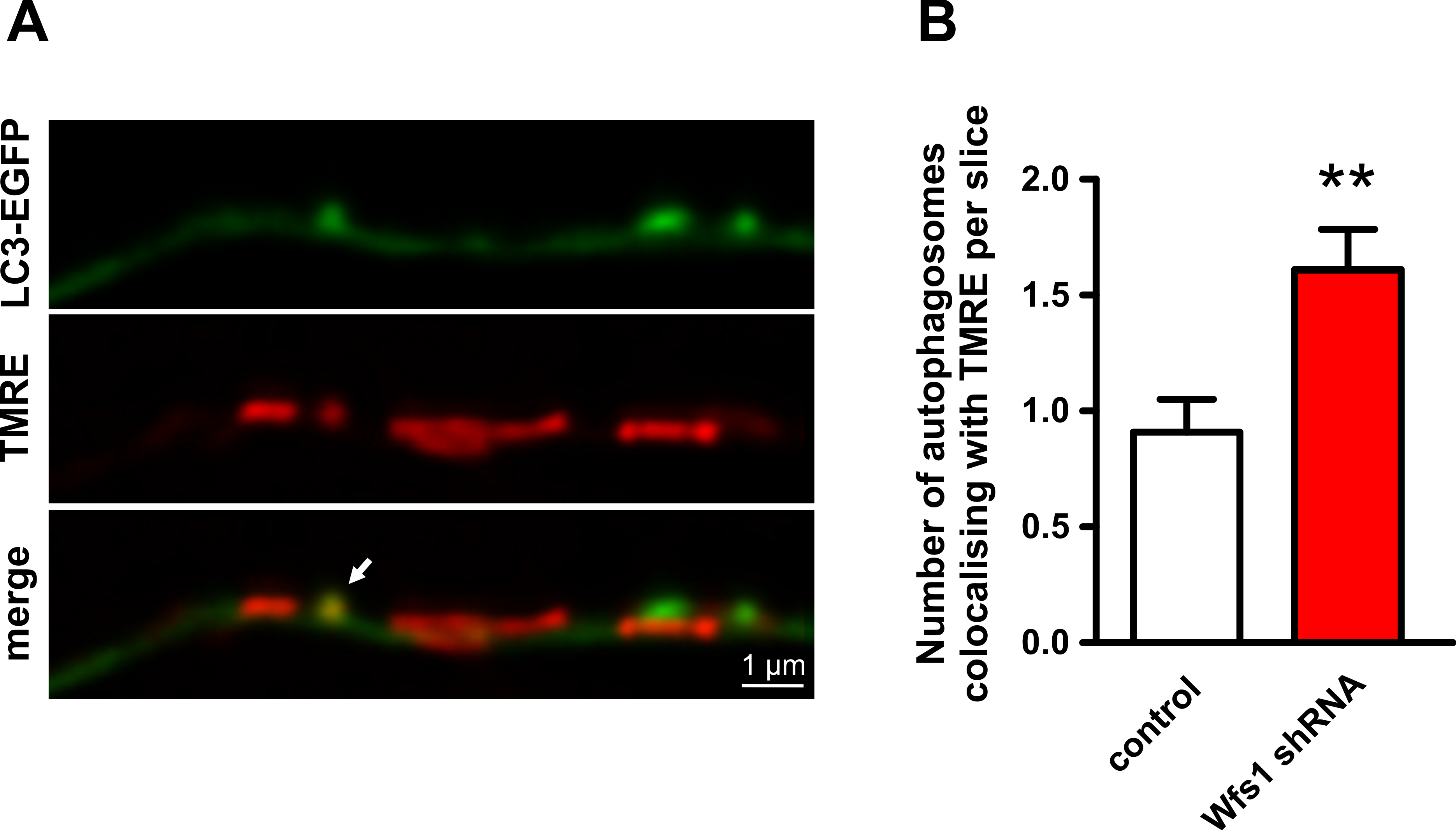

Supplement: S8 Fig — (A) Example of a TMRE-positive mitochondrion surrounded by an EGFP-LC3-positive autophagosome (indicated by arrow) in a Wfs1-shRNA transfected neuron. (B) Quantification of autophagosomes colocalising with TMRE per optical slice in the neuronal body. **p < 0.01 compared with control group. Underlying data is shown in S1 Data. (TIF) [file pbio.1002511.s009.tif]

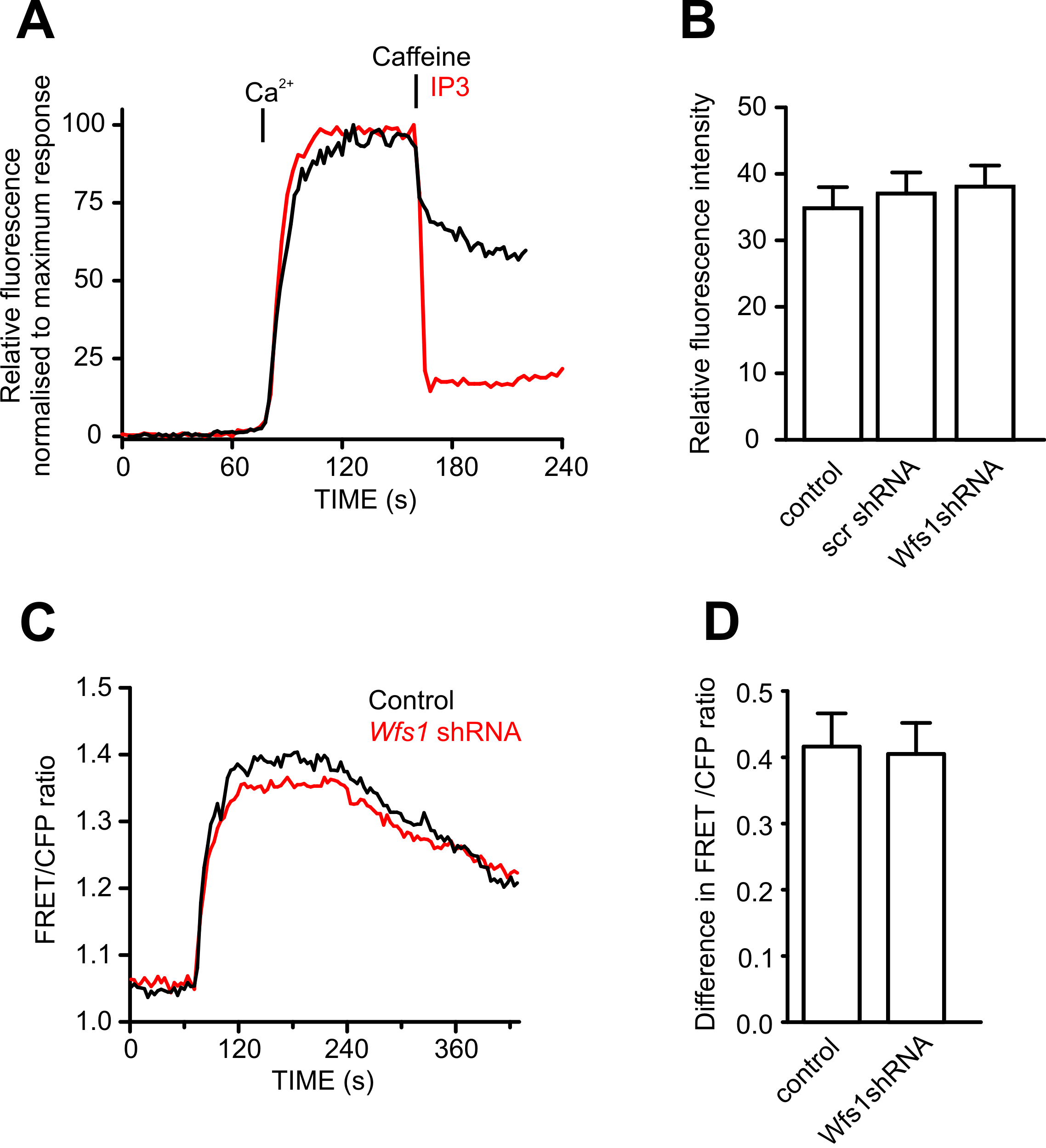

Supplement: S9 Fig — (A,B) Neurons transfected with scrambled shRNA or Wfs1 shRNA were permeabilised, loaded with the intra-reticular Ca2+ sensor Mag-fluo-4, and incubated with 100 nM Ca2+. IP3 (100 nM) induces much stronger ER Ca2+ release than caffeine (5 mM) (A). Maximal values of Ca2+-dependent ER fluorescence after incubation with 100 nM Ca2+ (B). (C–D) Cytosolic Ca2+ transients (C) were elicited by 30 μM cyclopiazonic acid (CPA; blocking ER Ca2+ uptake) in neurons transfected with the FRET-based cytosolic Ca2+ sensor cytoD3cpv and scrambled shRNA or Wfs1 shRNA. No difference was observed in the amplitude of Ca2+ transient (D). (TIF) [file pbio.1002511.s010.tif]

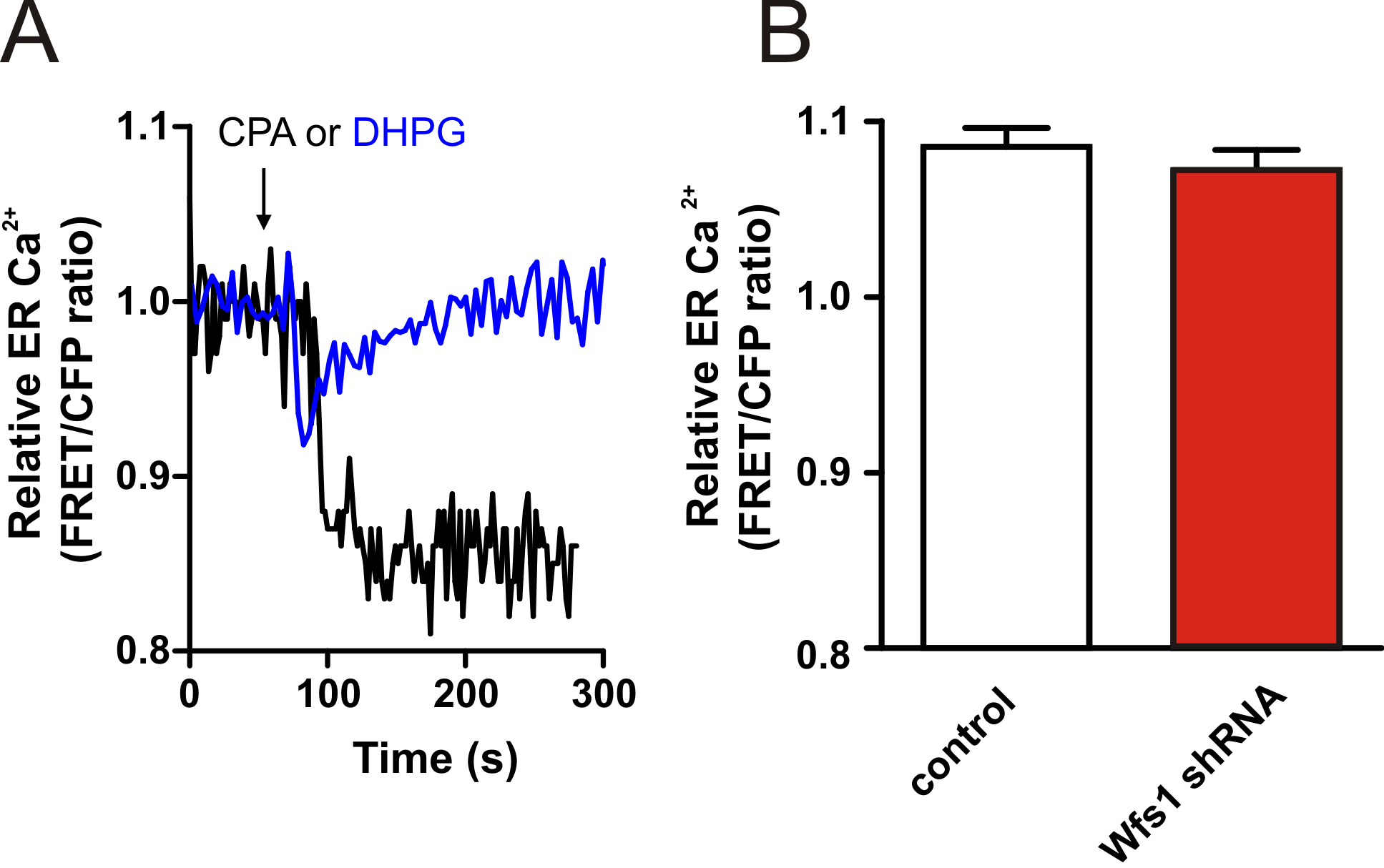

Supplement: S10 Fig — (A) Neurons were transfected with the ER-targeted second-generation Ca2+ sensor cameleon (D1ER) and treated with 30 μM CPA (blocking ER Ca2+ uptake, black line) or 200 μM DHPG (generating IP3, blue line) to induce a decrease in ER Ca2+ content. This experiment was performed to demonstrate that the probe responds to treatments decreasing ER Ca2+ levels. (B) Neurons were transfected with D1ER and scrambled or Wfs1 shRNAs. The results show no difference between control and WFS1-deficient neurons. Underlying data is shown in S1 Data. (TIF) [file pbio.1002511.s011.tif]

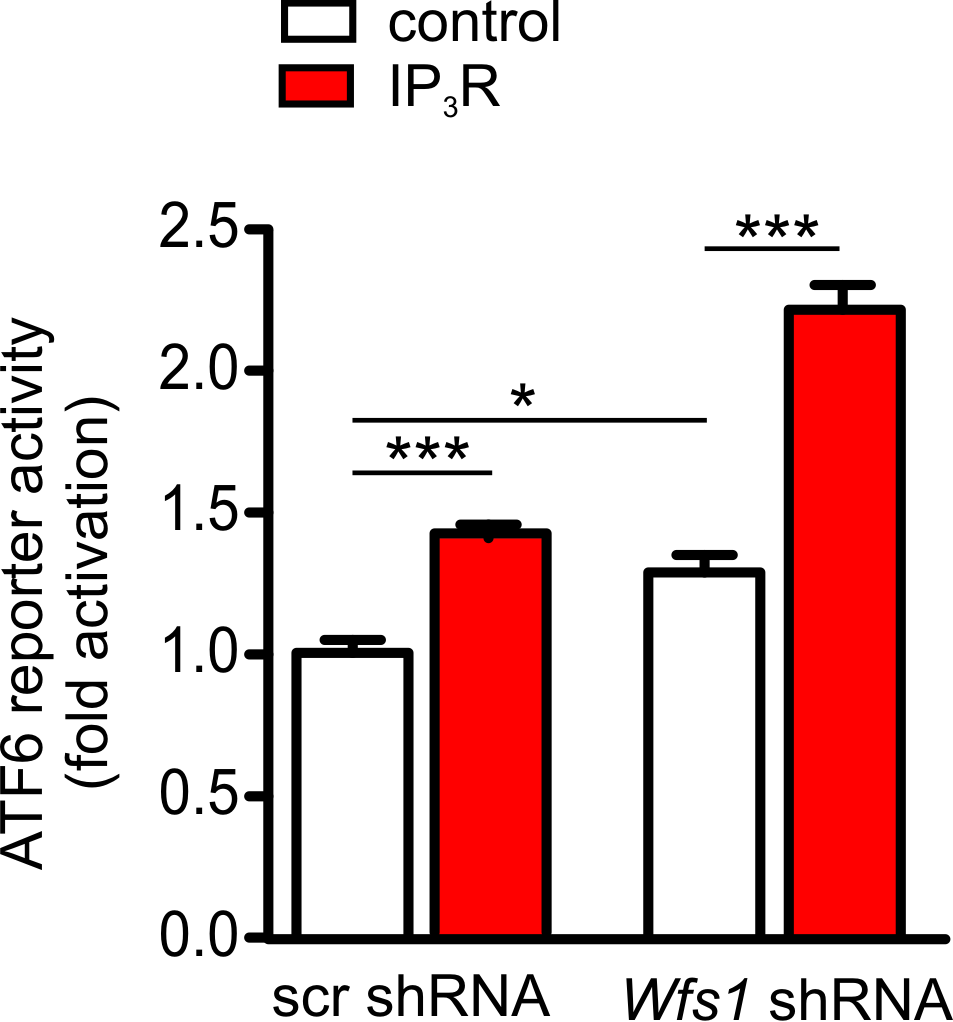

Supplement: S11 Fig — Neurons were transfected with plasmids expressing an ATF6 firefly luciferase reporter, Renilla luciferase, and scrambled shRNA or Wfs1 shRNA with or without IP3R. Indeed, overexpression of IP3R increases rather than supresses ATF6 reporter luminescence. *p < 0.05 and ***p < 0.001 versus indicated group. Underlying data is shown in S1 Data. (TIF) [file pbio.1002511.s012.tif]

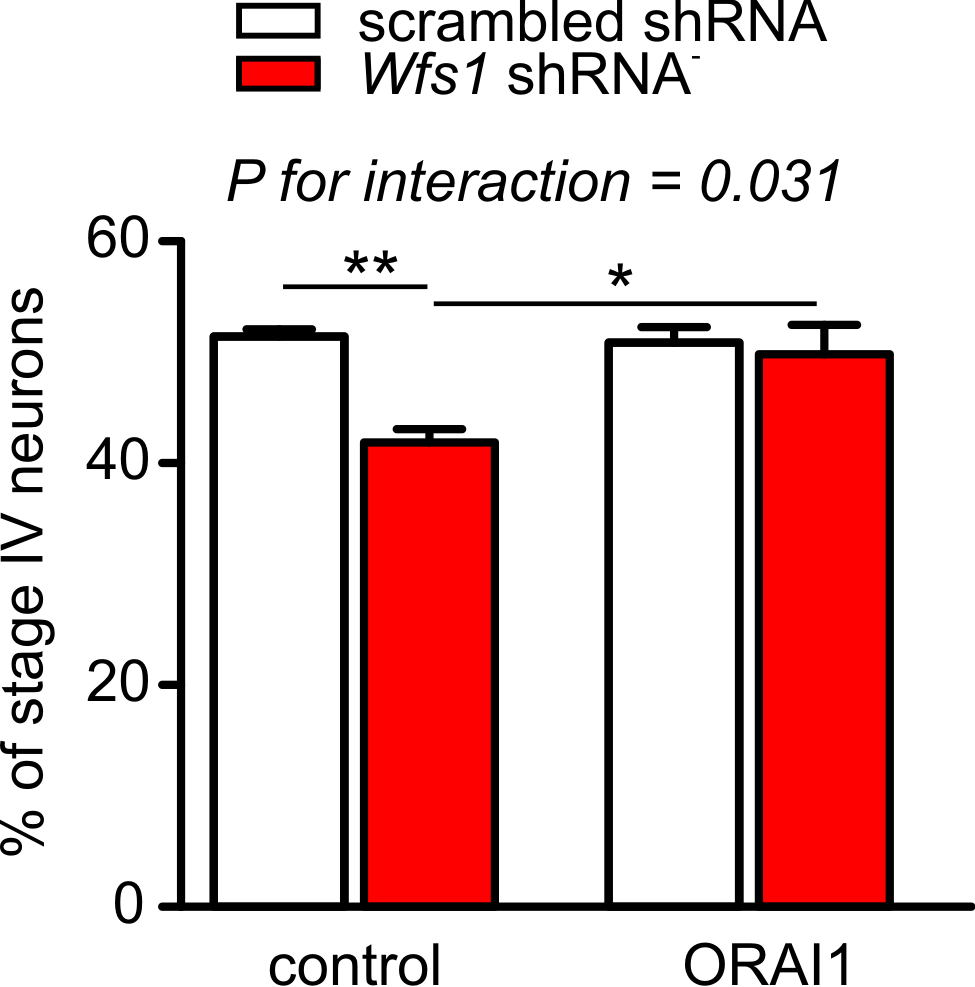

Supplement: S12 Fig — Neurons were transfected with the neuronal marker pAAV-hSyn-DsRed1 and scrambled shRNA or Wfs1 shRNA with or without ORAI1 at DIV1. Overexpression of ORAI1 restores the percentage of mature neurons in WFS1-silenced neurons, estimated at DIV4. *p < 0.05 and **p < 0.01 compared with indicated groups. Two-way ANOVA, p = 0.031 for interaction. Underlying data is shown in S1 Data. (TIF) [file pbio.1002511.s013.tif]

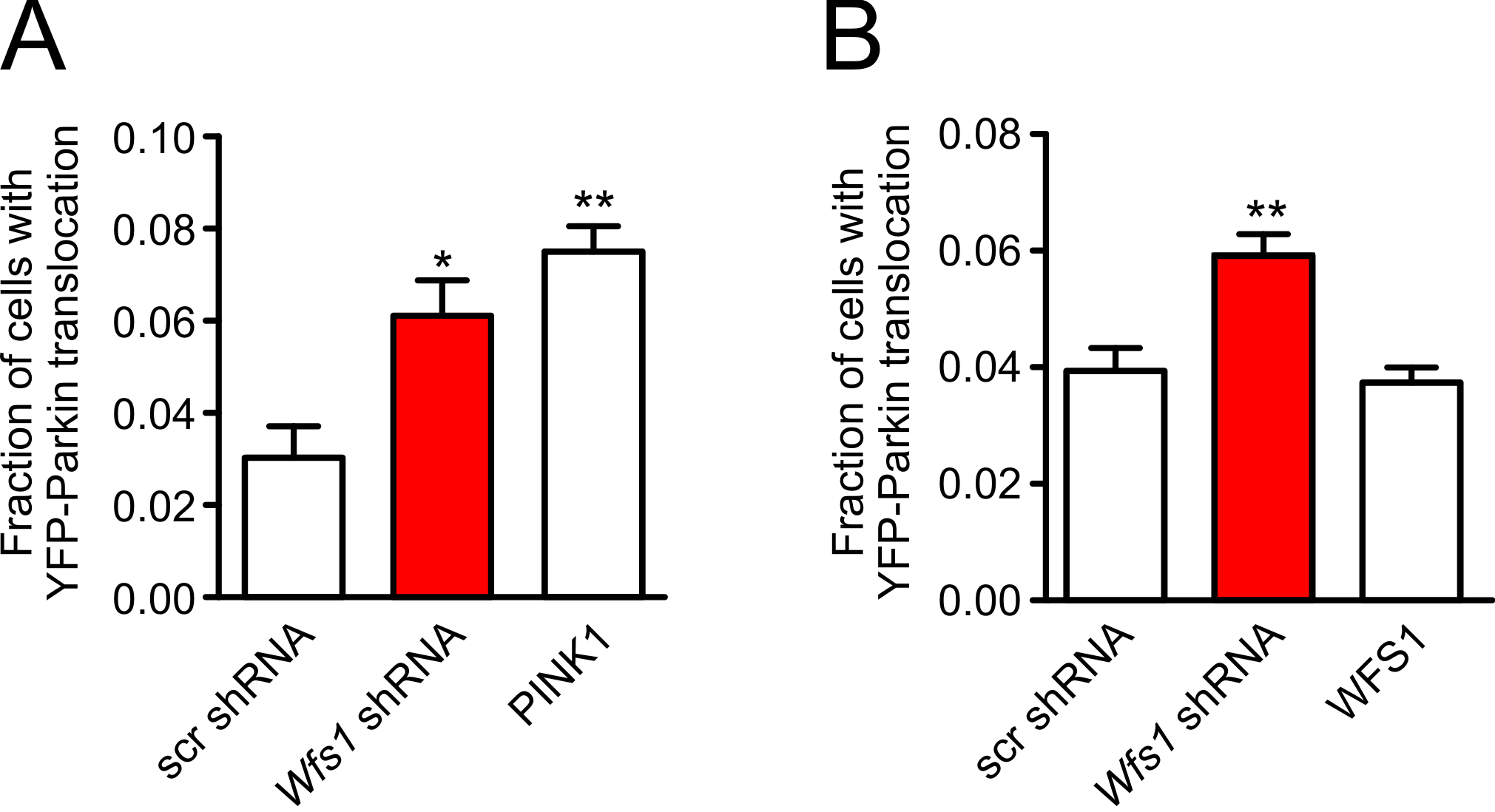

Supplement: S13 Fig — (A) PC12 cells were transfected with YFP-Parkin and scrambled shRNA, Wfs1 shRNA, or wt PINK1 and visualised 6 d later. The fraction of cells with YFP-Parkin translocated to mitochondria is higher in the Wfs1 shRNA-expressing group and in the PINK1-overexpressing group, used as a positive control. Underlying data is shown in S1 Data. (B) PC6 cells were transfected with YFP-Parkin and scrambled shRNA, Wfs1 shRNA, or wt WFS1 and visualised 5 d later. The fraction of cells with YFP-Parkin translocated to mitochondria is higher in the Wfs1 shRNA-expressing group. *p < 0.05 and **p < 0.01 compared with the scrambled shRNA group. (TIF) [file pbio.1002511.s014.tif]

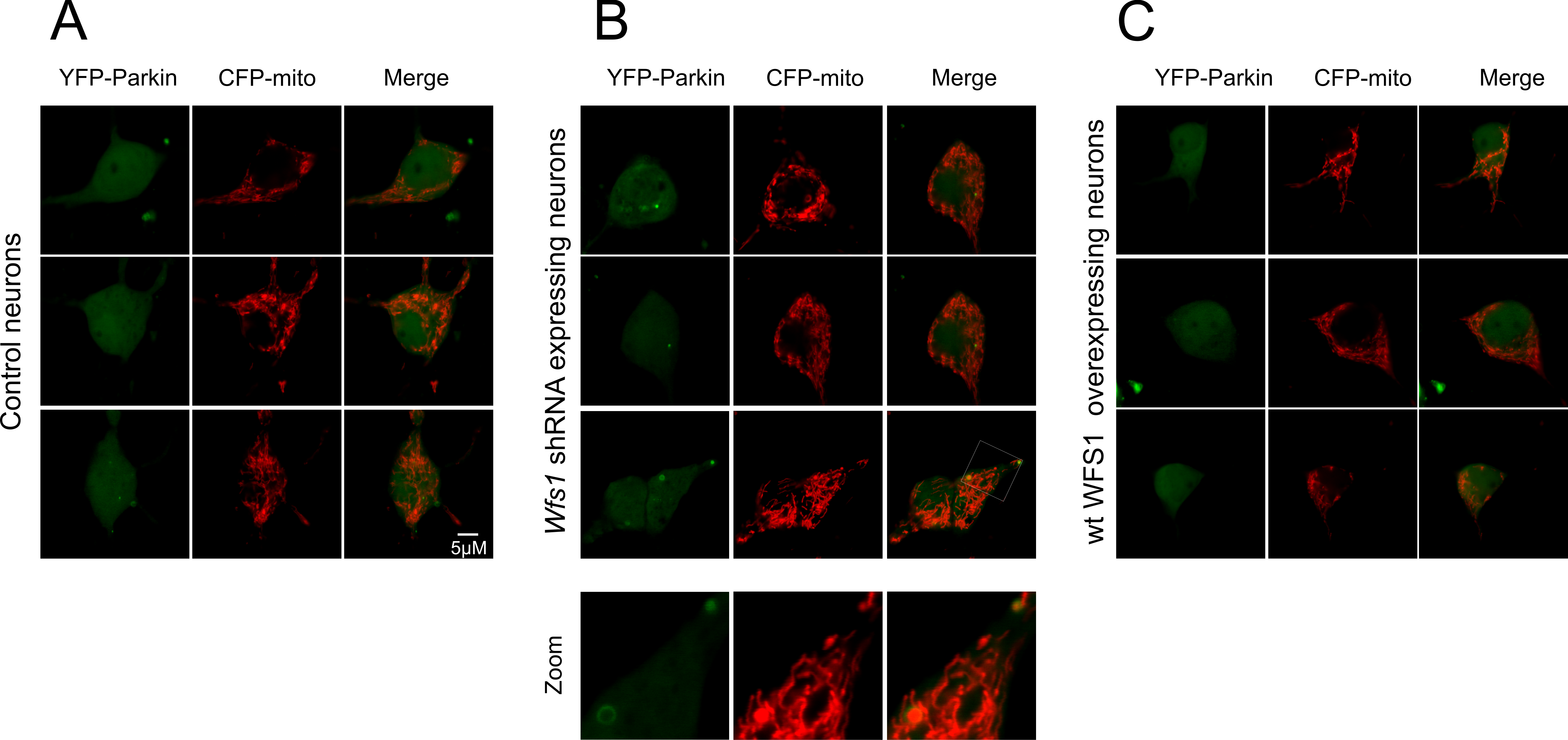

Supplement: S14 Fig — Notice the increased aggregation of YFP-parkin in Wfs1 shRNA expressing neurons. (TIF) [file pbio.1002511.s015.tif]

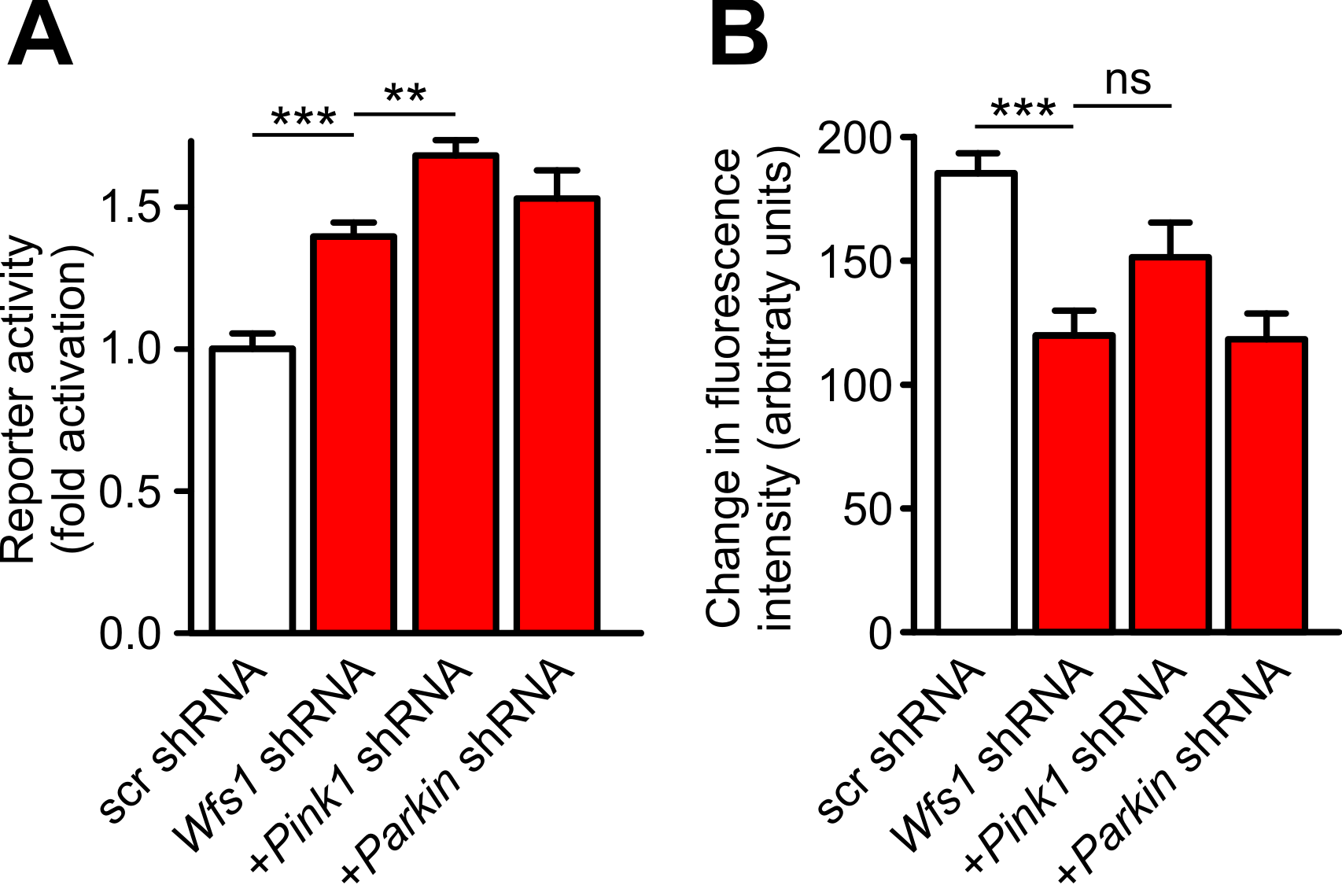

Supplement: S15 Fig — (A) Neurons were transfected with plasmids expressing ATF6 firefly luciferase reporter, Renilla luciferase, and scrambled shRNA or Wfs1 shRNA. Additional co-transfection with PINK1 shRNA or Parkin shRNA does not attenuate ATF6 activation induced by Wfs1 deficiency. (B) Neurons transfected with scrambled shRNA or Wfs1 shRNA were co-loaded with cytosolic Ca2+ sensor Fluo-4. Transfected cells were visualised by co-transfection with the mitochondrial marker mKate2-mito. PINK1 and Parkin silencing does not normalise the lowered amplitude of KCl-induced Ca2+ transients associated with WFS1 deficiency. **p < 0.01 and **p < 0.01 compared with indicated groups. Underlying data is shown in S1 Data. (TIF) [file pbio.1002511.s016.tif]

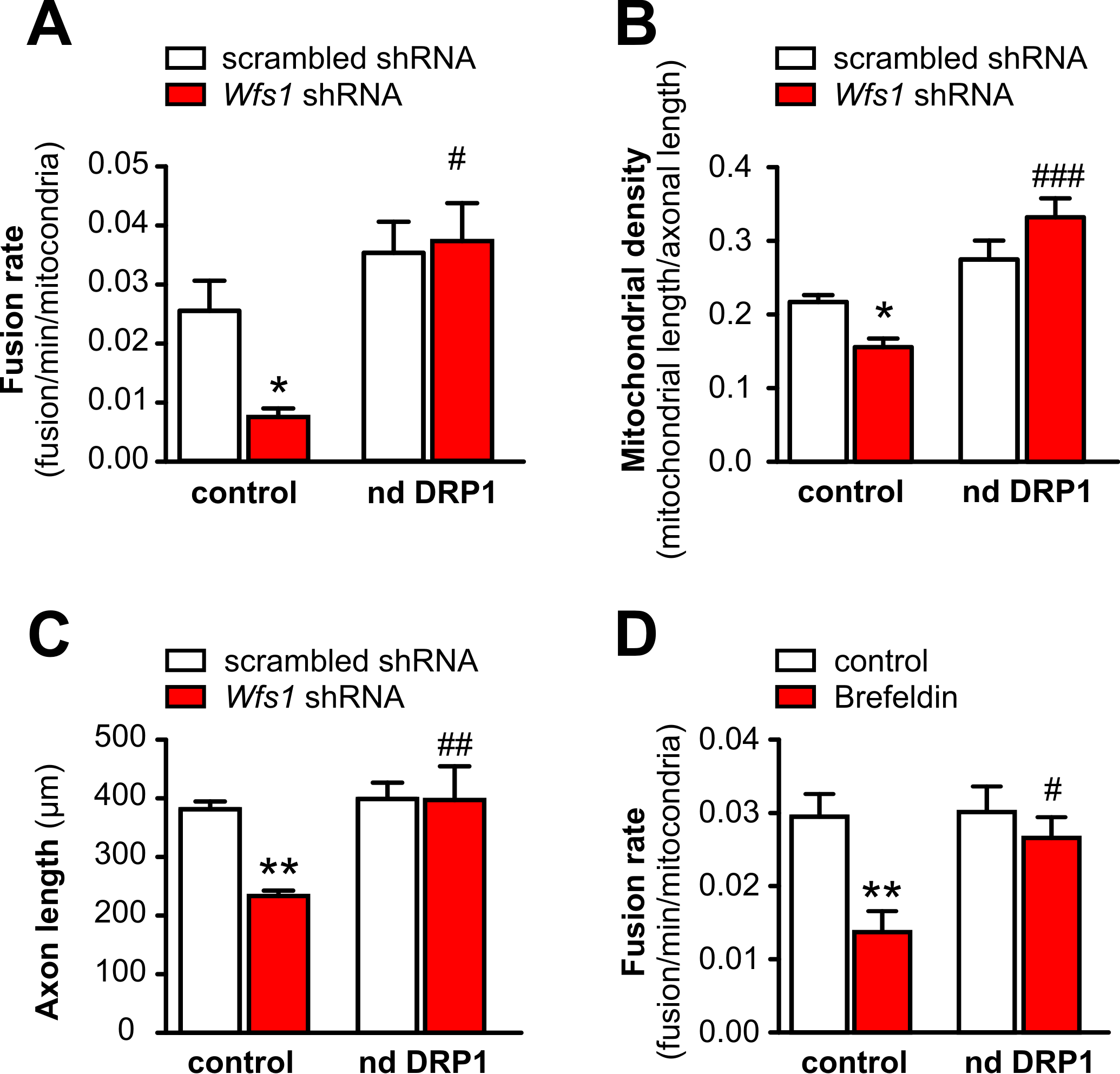

Supplement: S16 Fig — (A–C) Under conditions of WFS1 deficiency, nd DRP1 expression normalises mitochondrial fusion rate (A), mitochondrial density (B), and axon lengths (C). (D) nd DRP1 prevents Brefeldin-induced (5 μM for 48 h) inhibition of mitochondrial fusion rate. *p < 0.05 and **p < 0.01 compared with respective scrambled shRNA groups and #p < 0.05, ##p < 0.01, and ### p < 0.001 compared with respective Wfs1 shRNA groups. Underlying data is shown in S1 Data. (TIF) [file pbio.1002511.s017.tif]

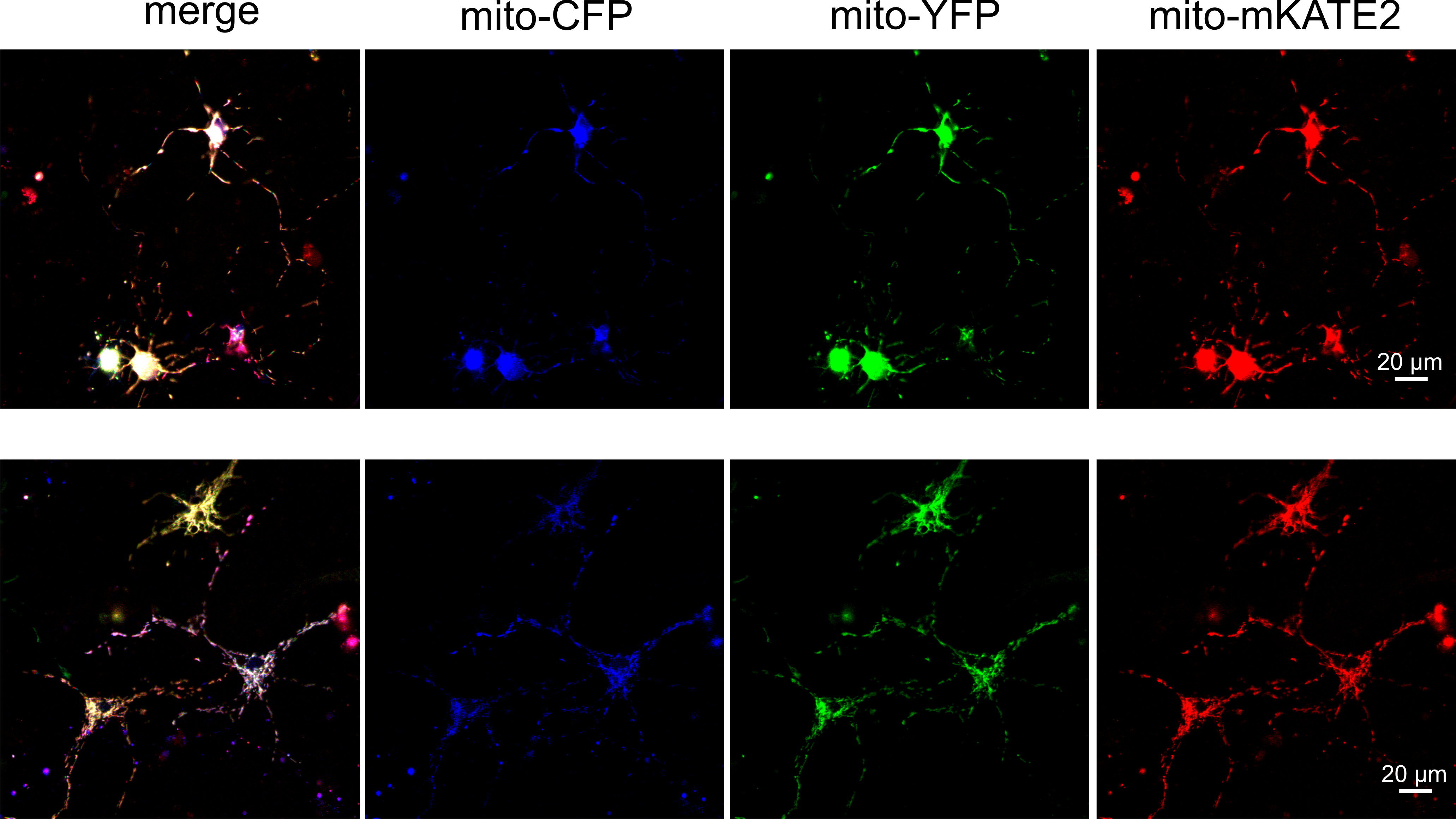

Supplement: S17 Fig — (TIF) [file pbio.1002511.s018.tif]

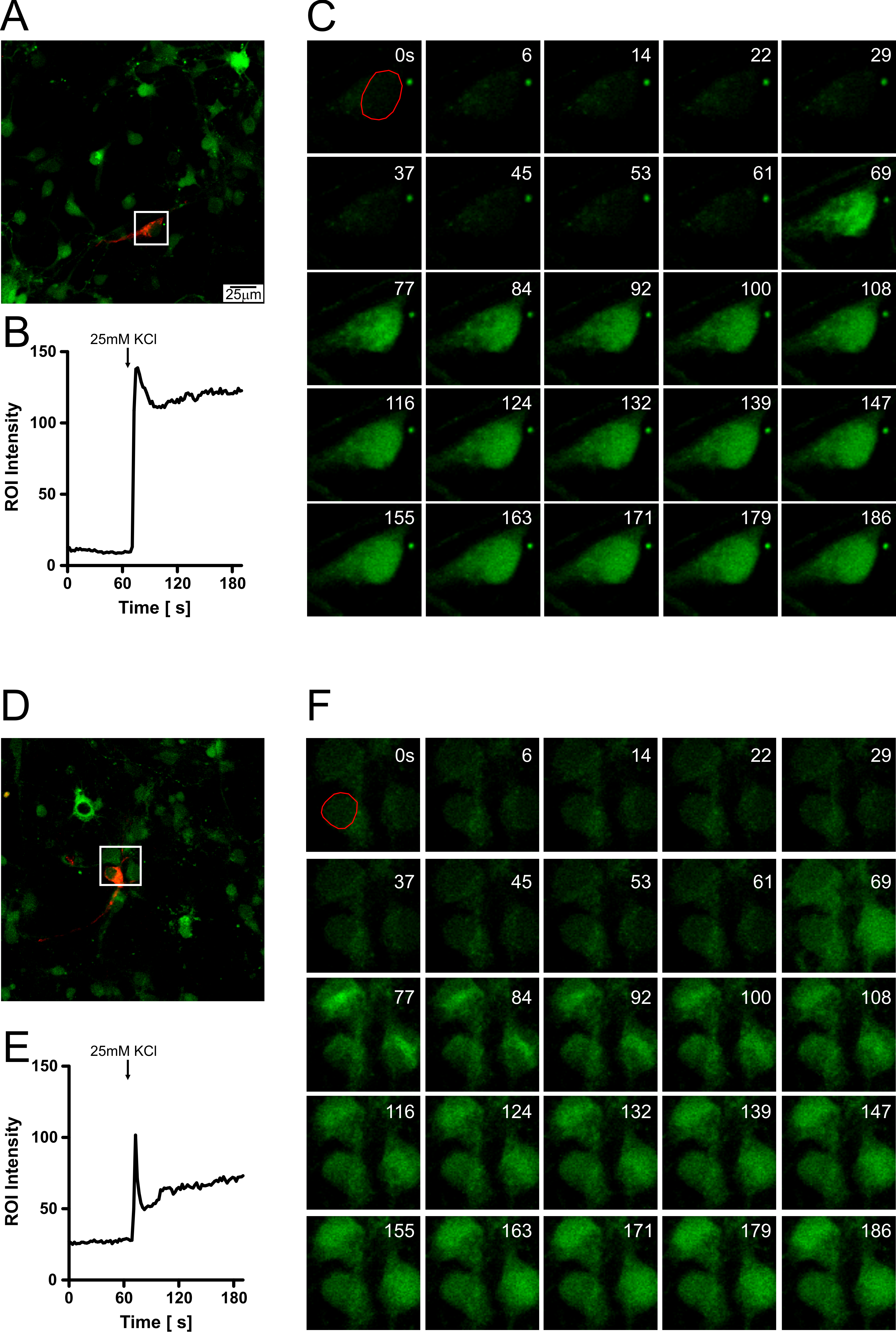

Supplement: S18 Fig — (A–C) Neurons transfected with the mitochondrial marker mKate2-mito and scrambled shRNA were loaded with the Ca2+ sensor Fluo-4. (A) A single image of an mKate2-mito-positive neuron marked with a white box. (B) A time-lapse series of images of a transfected neuron (KCl was added at 63 s). (C) ROI intensity during this time-lapse. The ROI is visible in the first image of the time lapse series. (D–F) Neurons transfected with the mitochondrial marker mKate2-mito and Wfs1 shRNA were loaded with the Ca2+ sensor Fluo-4. (D) A still image of an mKate2-mito-positive neuron marked by white box. (E) A time lapse series of images of a transfected neuron. (F) ROI intensity throughout the time-lapse. The ROI is visible in the first image of the time lapse series. Underlying data is shown in S1 Data. (TIF) [file pbio.1002511.s019.tif]
