# Supplementary material for: Role of Mitochondrial Dynamics in Neuronal Development: Mechanism for Wolfram Syndrome
Source: PLoS Biol. 2016 Jul 19;14(7):e1002511. doi: 10.1371/journal.pbio.1002511 (PMC4951053; doi:10.1371/journal.pbio.1002511)
Supplement: S1 Table — (DOCX) [file pbio.1002511.s020.docx]

| **scrambled shRNA** | | | | | |
| --- | --- | --- | --- | --- | --- |
|  |  |  |  |  |  |
|  | **I** | **II** | **III** | **IV** | **total** |
| **DIV2** | 0,037 | 0,604 | 1,103 | 0,045 | 1,789 |
| **DIV4** | 0,078 | 0,244 | 0,667 | 1,133 | 2,122 |
| **DIV6** | 0,000 | 0,111 | 0,333 | 1,578 | 2,022 |
| **DIV8** | 0,000 | 0,011 | 0,078 | 1,589 | 1,678 |
| ***Wfs1* shRNA** | | | | | |
|  | **I** | **II** | **III** | **IV** | **total** |
| **DIV2** | 0,117 | 0,873 | 0,710 | 0,000 | 1,700 |
| **DIV4** | 0,111 | 0,544 | 0,922 | 0,622 | 2,200 |
| **DIV6** | 0,000 | 0,144 | 0,411 | 1,611 | 2,167 |
| **DIV8** | 0,000 | 0,000 | 0,078 | 1,356 | 1,433 |
