# Supplementary material for: Role of Mitochondrial Dynamics in Neuronal Development: Mechanism for Wolfram Syndrome
Source: PLoS Biol. 2016 Jul 19;14(7):e1002511. doi: 10.1371/journal.pbio.1002511 (PMC4951053; doi:10.1371/journal.pbio.1002511)
Supplement: S2 Table — (DOCX) [file pbio.1002511.s021.docx]

| **Experiment type** | **Replicate** | **n** | **Explanation** |
| --- | --- | --- | --- |
| Fusion rate | dish | ≥ 4 | Data from ≥ 4 axons (exceptionally from 3) were pooled within each dish, a mean of dishes/treatment generated and used for comparison |
| Mitochondrial density | axon | ≥ 16 | Collected from ≥ 4 dishes/group (at least 4 axons per dish) |
| Mitochondrial length | axon or mitochondrion | ≥ 16 axons or  ≥ 150 mito-chondria | Collected from ≥ 4 dishes/group (at least 4 axons per dish) |
| Mitophagy (Keima) | neuron | ≥ 30 | Collected from ≥ 3 dishes/group (10 neurons per dish) |
| Mitophagy (LC3) | neuron | 50 | Collected from 5 dishes/group (10 neurons per dish) |
| ATP | neuron | 60 | Collected from 5 dishes/group (10 neurons per dish) |
| Membrane potential (JC10) | dish | 7 | Eight fields were pooled for each dish, a mean of dishes/treatment was generated and used for comparison |
| Membrane potential (TMRE) | dish | 10 dishes | Ten fields were pooled for each dish, a mean of dishes/treatment was generated and used for comparison |
| Cytosolic calcium transients (Fluo-4) | neuron | ≥ 8 | 8-12 (exceptionally 6-38) neurons collected from at least 4 dishes/group |
| Cytosolic calcium transients (FRET) | neuron | 16-19 | Collected from 8 dishes/group |
| Cytosolic baseline calcium | neuron | 40 | Collected from 4 dishes/group |
| ER baseline calcium | neuron | 89-90 | Collected from 7 dishes/group (at least 10 neurons per dish) |
| Cytosolic calcium measurements (aequorin) | dish | 24-25 | From 6 independent sister cultures (3-5 dishes per culture) |
| Luciferase reporter assays | well | ≥10 | Luminescence from ≥10 wells of 96 well plate (with exception of positive controls) |
| Development | dish | 3 | 30 fields were pooled for each dish, a mean of dishes was generated and used for comparison |
| Axonal growth | neuron | 18-40 | Collected from ≥ 3 dishes/group (6-12 neurons per dish) |
| Synapse density | axon | 24-48 | Collected from 4-5 dishes/group |
| MRI | brain | 4 | Control and Wfs1 knockout age-matched mice |
| RT-PCR | brain | 4-6 | Control and Wfs1 knockout aged-matched mice |
| Mitochondrial motility | mitochondrion | 327- 383 | Collected from 20 axons from 5 individual dishes (4 axons per dish) per group |
| Parkin translocation | dish | 4-6 | 10 different fields were pooled per dish |
| Neuronal survival | dish | 61 | Field averages from 61-62 individual dishes from 17 independent sister cultures |
